# Akt-mediated Ephexin1–Ras interaction promotes oncogenic Ras signaling and colorectal and lung cancer cell proliferation

**DOI:** 10.1038/s41419-021-04332-0

**Published:** 2021-10-28

**Authors:** Jeeho Kim, Young Jin Jeon, Sung-Chul Lim, Joohyun Ryu, Jung-Hee Lee, In-Youb Chang, Ho Jin You

**Affiliations:** 1 Laboratory of Genomic Instability and Cancer therapeutics, Chosun University School of medicine, 375 Seosuk-Dong, Gwangju, 501-759 South Korea; 2 Department of Pharmacology, Chosun University School of medicine, 375 Seosuk-Dong, Gwangju, 501-759 South Korea; 3 Department of Pathology, Chosun University School of medicine, 375 Seosuk-Dong, Gwangju, 501-759 South Korea; 4 The Hormel Institute, University of Minnesota, 801 16th Avenue NE, Austin, MN 55912 USA; 5 Department of Cellular and Molecular Medicine, Chosun University School of medicine, 375 Seosuk-Dong, Gwangju, 501-759 South Korea; 6 Department of Anatomy, Chosun University School of medicine, 375 Seosuk-Dong, Gwangju, 501-759 South Korea

**Keywords:** Targeted therapies, Cancer models

## Abstract

Ephexin1 was reported to be highly upregulated by oncogenic Ras, but the functional consequences of this remain poorly understood. Here, we show that Ephexin1 is highly expressed in colorectal cancer (CRC) and lung cancer (LC) patient tissues. Knockdown of Ephexin1 markedly inhibited the cell growth of CRC and LC cells with oncogenic Ras mutations. Ephexin1 contributes to the positive regulation of Ras-mediated downstream target genes and promotes Ras-induced skin tumorigenesis. Mechanically, Akt phosphorylates Ephexin1 at Ser16 and Ser18 (pSer16/18) and pSer16/18 Ephexin1 then interacts with oncogenic K-Ras to promote downstream MAPK signaling, facilitating tumorigenesis. Furthermore, pSer16/18 Ephexin1 is associated with both an increased tumor grade and metastatic cases of CRC and LC, and those that highly express pSer16/18 exhibit poor overall survival rates. These data indicate that Ephexin1 plays a critical role in the Ras-mediated CRC and LC and pSer16/18 Ephexin1 might be an effective therapeutic target for CRC and LC.

## Introduction

Oncogenic Ras mutations are frequently observed in solid tumor cells and are present in pancreatic cancer, colorectal cancer (CRC), and lung cancer (LC) [1, 2]. Mutational activation of K-Ras in these tissues is sufficient to initiate neoplasia in mice [3–5]. Thus, understanding the mechanisms behind Ras-induced oncogenesis is an important goal in cancer therapy. A significant consequence of Ras-mediated signal transduction is the altered expression of a large number of genes. It is well established that canonical Ras signaling is coupled to transcriptional regulation through the activation of the MAPK cascade, which involves the sequential phosphorylation and activation of the serine/threonine kinases RAF, MEK1/2, and ERK1/2 [6–12]. More recently, new regulatory factors that directly interact with K-Ras were identified and shown to play a crucial role in the full range of K-Ras oncogenic phenotypes [13–19]. Despite decades of effort, many aspects of the molecular mechanism underlying Ras-induced tumorigenesis and possible therapeutic targeting remain difficult to identify.

Ephexin1 is a member of the DbI family of guanine nucleotide exchange factors (GEFs) and serves as a direct link between Eph receptors and the Rho-family of GTPases [20, 21]. Ephexin1 is highly expressed in the nervous system during development and is involved in many neurophysiological events [22–26]. It is phosphorylated by the Src family of kinases and by fibroblast growth factor receptor signaling, leading to the regulation of GEF activity, which in turn regulates actin cytoskeletal dynamics [22, 27]. Through extensive analysis of the gene expression patterns in cells with activated forms of Ras and Rho families, it was found that Ephexin1 was highly upregulated by H-Ras^G12V^ in NIH3T3 cells [28]. In humans, Ephexin1 is upregulated in papillary thyroid cancers (PTC) and has been identified as the most reliable marker for PTC diagnosis [29]. Therefore, the implication of these results is that Ephexin1 may be involved in tumor proliferation and progression, particularly in cancer cells with oncogenic Ras mutations. However, the functional consequences of Ephexin1 had not yet been addressed.

In the present study, we show that Ephexin1 is overexpressed in both CRC and LC tissues and is associated with a poor prognosis for both cancers, and provide evidence for the functional and clinical significance of Ephexin1 and identify a potential therapeutic target for the case of CRC and LC caused by Ras mutations.

## Methods

### Cell culture and chemicals

Normal lung cells (IMR90, MRC5, WI38) and normal colon cells (CCD 18co, CCD 841coN) were cultured in MEM medium (Invitrogen, Carlsbad, CA, USA). LC cells (A549, H23, H358, H1299, H1666, H460, and H1650) and colon cancer cells (LoVo, HCT15, SW480, KM12C, and KM12SM) were grown in RPMI-1640 medium (Invitrogen). SK-MES-1 and Calu-3 (LC) and Caco-2 and LS174T (colon cancer) cells were cultured in MEM medium. HEK293T (human embryonic kidney), SW620, DLD-1, and HT-29 (colon cancer) cells were maintained in Dulbecco’s Modified Eagle Medium (Invitrogen). HCT116 (colon cancer) cells were cultured in an IMDM medium (Invitrogen), respectively. All cell lines were purchased from the American Type Culture Collection (ATCC, Manassas, VA, USA). All media were supplemented with 10% fetal bovine serum and 1% penicillin/streptomycin antibiotic solution. Cells were maintained in 5% CO_2_ in a humidified atmosphere at 37 °C. Plasmids were transiently transfected into mammalian cells using TurboFect (Thermo Scientific, Waltham, MA, USA). Cycloheximide and EGF were purchased from Sigma-Aldrich (St Louis, MO, USA) and crystal violet was obtained from Samchun Chemical (Pyeongtaek, South Korea). LY24002, PD98059, U0126, and SB202190 were purchased from EMD Biosciences (Gibbstown, NJ, USA). AZD 5363 was purchased from Tocris Bioscience (Ellisville, USA).

### Plasmid constructs and cloning

Human Ephexin1 cDNA was amplified from HEK293T cells by reverse transcription-polymerase chain reaction (RT-PCR) and cloned into the pCI-neo-Flag or pCI-neo-V5 mammalian expression vectors (Promega, Madison, WI, USA). To prepare serial deletion constructs of Ephexin1 (∆SH3 [1–612], ∆PH/SH3 [1–489], RR [1–273], DH/PH [273–601], ∆DH [1–273,489–710], ∆PH [1–457,612–710], ∆DH/PH [1–273,612–710], ∆RR [273–710], DH/PH [273–546], DH [273–489], DH [273–395], DH/PH [334–610], DH/PH [395–610], PH [457–601], DH/PH [395–546]), each fragment was PCR-amplified using pCI-neo-V5-Ephexin1 as template, and the PCR products were inserted into Xho1 and Not1 sites of pCI-neo-V5 vector (all amino acid positions were based on the sequence of Ephexin1 with the accession number NM_019850).

For in vitro GST-pull down assay, Ephexin1 DH/PH domain and K-Ras constructs were cloned into pGEX4T-1 (GE Healthcare, Fairfield, CT, USA) and pET-28c (+) vectors (Novagen, Darmstadt, Germany), respectively. A comprehensive list of all PCR primers used in this study can be found in Supplementary Table S1. The pCI-neo-Flag-Ephexin1mutants (S16A/S18A, S16D/S18D, and S16E/S18E), pUSE-Myc-Akt-KD (K179M), pMEV-2xHA-K-Ras2B (Q61L), and Ephexin1 shRNA resistance pCI-neo-Flag-tagged WT and mutants (S16A, S18A, S606A, S16A/S18A, S16D/S18D, and S16E/S18E) Ephexin1 constructs were generated by site-directed mutagenesis (Quikchange II Site-Directed Mutagenesis^®^ kit, Agilent Technologies, Santa Clara, CA, USA). PCR primer sequences for site-directed mutagenesis are listed in Supplementary Table S2. For Ephexin1 single-guide RNA (sgRNA), sgRNA were designed using CRISPR RGEN Tools (http://www.rgenome.net/cas-designer/). Ephexin1 sgRNA specifically recognized the Exon 5 of Ephexin1 gene. Ephexin1 sgRNA was selected by the out-of-frame score of 68.8. Ephexin1 sgRNA sequence is 5′-TTACTGAGCAGGCAGATCTGGGG-3′. This sgRNA was inserted into BbsI sites of pX458 vector (Addgene: #48138). All constructs were verified by sequencing. pMEV-2xHA-K-Ras2B^G12V^ and pMEV-2xHA-K-Ras2B^S17N^ were purchased from Biomyx (San Diego, CA, USA), pGEX2TK-Pak1 (#12217), pGEX-2T-Rhotekin (#15247), and pGEX-2T-Raf-1 (#13338) plasmids were obtained from Addgene (Watertown, MA, USA), and pUSE-Myc-AKT-WT and pUSE-Myc-AKT-myr plasmids were procured from Upstate Biotechnology (Thermo Scientific).

### Quantitative RT-PCR (RT-qPCR)

Total RNA was extracted from cell lysates using TriZol (Invitrogen), and 2 μg of total RNA was reverse transcribed to cDNA using an oligo dT primer and M-MuLV Reverse Transcriptase (Invitrogen). RT-qPCR analysis was performed using specific primers and the SYBR Premix Ex Taq^™^ kit (TaKaRa Bio, Shiga, Japan). The transcripts were detected by CFX96 Real-Time PCR Detection System (BioRad, CA, USA). Primers used for RT-qPCR were Ephexin1, LPAR1, CTNNA2, SOX9, CDR1, CCND2, TSPAN7, CXCR4, LMO3, FZD3, TERT, SMPDL3B, IF144L, PTPRU, IL12RB2, APOL3, MAFB, MX1, BCL11B, and β-actin. Each sample was analyzed in triplicates, and target genes were normalized relative to the reference housekeeping gene, β-actin. Relative mRNA expression levels were calculated using the comparative threshold cycle (*C*_t_) method with β-actin as the control, according to the formula: Δ*C*_t_ = *C*_t_ (β-actin) − *C*_t_ (target gene). The fold change in gene expression normalized to β-actin and relative to the control sample was calculated as $2^{-\Delta\Delta C_{{\rm{t}}}}$. RT-qPCR primer sequences are listed in Supplementary Table S3.

### RNAi and generation of stable Ephexin1 knockdown cells

Cells were transfected with siRNAs (40 nM) using Lipofectamine 2000 (Invitrogen). After 36 h, cells were trypsinized, re-plating, and transfected again for another 36 h. Knockdown efficiencies were verified by western blot analysis. The sequences of Ephexin1 siRNA and shRNA can be found in Supplementary Table S4. HCT116, H1299, H460, and A549 cells were transfected with pSilencer2.1-U6-hygro control shRNA or pSilencer2.1-U6-hygro Ephexin1 shRNA using lipofectamine 2000 (Invitrogen) and cultured in a selection medium containing 500 μg/ml hygromycin for 4–5 weeks. After selection, stable Ephexin1 knockdown clones were confirmed by western blot analysis.

### Immunoblot and immunoprecipitation analysis

Cell extracts were prepared in RIPA buffer (50 mM Tris-HCl (pH 8.0), 150 mM NaCl, 1% Nonidet P-40, 0.5% sodium deoxycholate, 0.1% SDS) containing protease inhibitors (1 mM Na_2_VO_4_, 10 mM NaF, 2 mM PMSF, 5 μg/ml Leupeptin, 10 μg/ml Aprotinin, 1 μg/ml Pepstatin A) (Roche, Switzerland). Equal amounts of proteins were separated by sodium dodecyl sulfate-polyacrylamide gel electrophoresis (SDS-PAGE) followed by electrotransfer onto polyvinylidene fluoride membranes (PALL Life Sciences, USA), Membranes were subsequently incubated with appropriate primary antibodies overnight at 4 °C, followed by incubation with peroxidase-conjugated secondary antibodies for 1 h at room temperature. The bands were visualized by using the ECL chemiluminescent detection system (iNtRON Biotechnology). For immunoprecipitation of protein complexes, cell extracts were pre-cleared with protein G-Sepharose beads (GE Healthcare) and incubated with appropriate antibodies. The antibodies are listed in Supplementary Table S5. Antibody against S16 and S18 phosphorylated Ephexin1 was generated to phospho-peptide of Ephexin1 encompassing amino acids 11–23 (KTRRKpSApSDQWNT) using polyclonal antibody production service (Abfrontier, Seoul, South Korea).

### Bioinformatics analysis of RNA-Seq data in TCGA and GTEx

**Fig. 1:**
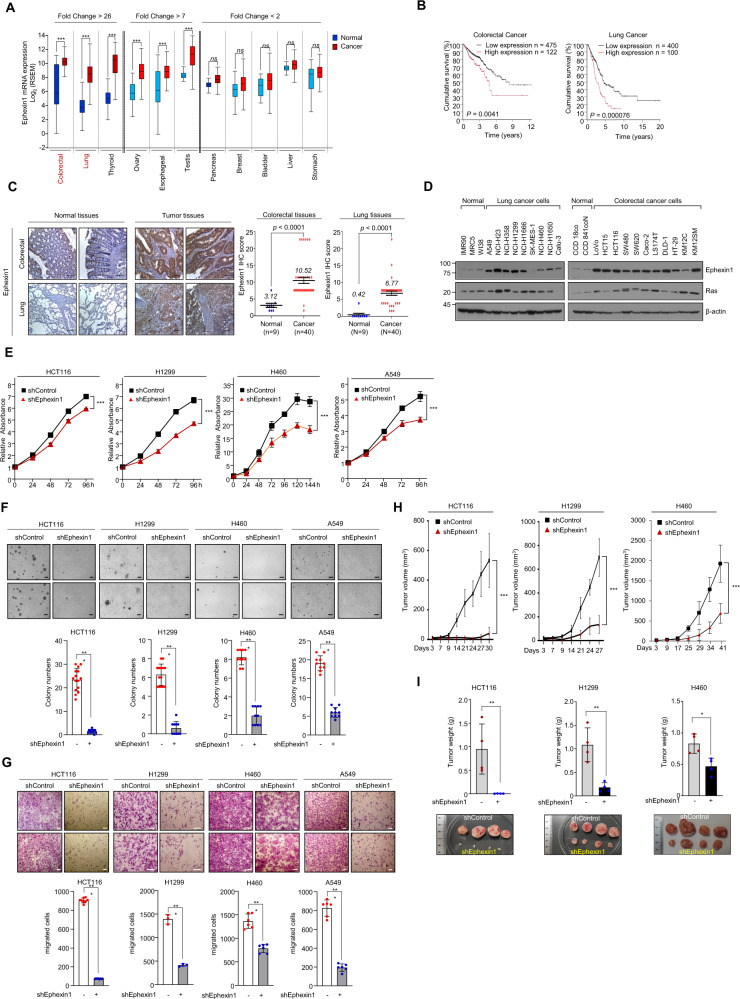
Ephexin1 is upregulated in CRC and LC and promotes tumor cell growth and proliferation. **A** Expression of Ephexin1 mRNA in 12 primary cancer types from TCGA. Data are shown as mean ± SD. ns not significant, ^***^P < 0.001, two-tailed Student’s *t* test. **B** Kaplan–Meier analysis of overall survival according to Ephexin1 in CRC and LC patients. *P* values are for a log-rank test. **C** Immunohistochemistry (IHC) staining was performed to evaluate Ephexin1 expression in CRC and LC tissues and their corresponding normal tissues. Hematoxylin is the counterstain. Scale bar = 100 μm. Data are shown as mean ± SD, *P* values are for a two-way ANOVA. **D** Ephexin1 protein expression in normal and indicated colon (*n* = 8) and lung (*n* = 11) cancer cell lines were examined by western blot. **E** Effect of Ephexin1 knockdown on cell proliferation by MTT assay in HCT116, H1299, H460, and A549 cells. Data are shown as mean ± SD. ^***^P < 0.001, two-way ANOVA. **F**, **G** Anchorage-independent growth assessed by soft agar assay (**F**) and migration ability (**G**) determined by transwell assay in Ephexin1-depleted HCT116, H1299, H460, and A549 cells. Representative images are shown. Scale bar = 100 μm. Data are shown as mean ± SD. ^**^P < 0.01, and ^***^P < 0.001, two-tailed Student’s *t* test. **H** Control and Ephexin1-depleted HCT116, H1299, and H460 cells were inoculated subcutaneously in BALB/C nude mice (*n* = 4). Tumor volumes recoded at the indicated times are shown. Data are shown as mean ± SD. ^***^P < 0.001, two-way ANOVA. **I** Average tumor weight of each group at the endpoint of the experiment are shown. Data are shown as mean ± SD. ^*^P < 0.05 and ^**^P < 0.01, two-tailed Student’s *t* test.

The Cancer Genome Atlas (TCGA; https://www.cancer.gov/about-nci/organization/ccg/research/structural-genomics/tcga) and Genotype-Tissue Expression (GTEx; https://commonfund.nih.gov/GTex) databases were downloaded using the UCSC Xena browser Data Hub (https://xenabrowser.net/hub/). RNA sequencing data as measured by Illumina HiSeq (RSEM normalized) were downloaded whenever available. The TCGA mRNA expression of the discovery set was transformed into a log_2_ scale. *P*-values between groups were calculated Student’s-test using GraphPad Prism (GraphPad Software Inc., CA, USA) (Fig. 1A). TCGA datasets contain survival data with clinical information and TCGA survival curves were visualization using The Human Protein Atlas (https://www.proteinatlas.org/). The cut-off value for dividing Ephexin1 high and low in TCGA data is FPKM (fragments per kilobase per millions mapped reads) = 8.5 (colorectal), FPKM = 3.6 (Lung) (Fig. 1B). Kaplan–Meier analysis was performed to estimate the survival curves of the different subgroups and the log-rank test was used to compare the curve.

### RNA sequencing analysis and GSEA

Total RNA was harvested directly from cell culture plates using 1 ml TRIzol reagent per 60 mm plate. The total RNA was isolated and treated with DNase I (Invitrogen) Total RNA sequencing was performed using an Illumina NovaSeq6000^™^ sequencer at the DNA_Link^™^ (Korea, Seoul). RNA-seq reads were first mapped to the human genome GRCh37/hg19 build using Tophat version 2.0.13 (http://ccb.jhu.edu/software/tophat/) [30]. The aligned results were added to Cuffdiff version 2.2.1 (http://cole-trapnell-lab.github.io/cufflinks/papers/) [31] to calculate FPKM (Fragments per Kilobase of transcript per Million) value and to report differentially expressed genes. For library normalization and dispersion estimation, geometric, and pooled methods (http://cole-trapnell-lab.github.io/cufflinks/cuffdiff/) were applied. We created a scatter plot and heatmap using the function “heatmap. 2” in “ggplot” package in R 3.4.1. The data discussed in this publication have been deposited in the NCBI Gene Expression Omnibus (GEO) and are accessible through GEO Series accession number GSE147809. Gene set enrichment analysis (GSEA) was carried out GSEA pre-ranked module on the GSEA software (version 4.0.3) [32, 33] with log_2_ fold change values for ranking genes.

### Cell growth assay

Cell growth assay was performed using the MTT assay. An equal number of HCT116, H1299, H460, and A549 cells were seeded in triplicate well in 48-well plates, at a density of 1 × 10 cells/0.2 ml/well [4]. Twenty microliters of MTT mixture (5.0 mg/ml) in the IMDM or RPMI1640 medium was added, and the plate was incubated for indicated times at 37 °C. The purple formazan crystals thus formed were dissolved in 200 µl of MTT solvent (0.1% NP-40 and 4 mM HCl in isopropanol), gently mixing at room temperature, and the optical densities of the wells on the plate were read at 570 nm using a microplate spectrophotometer (Epoch, BioTeck, Winooski, VT, USA).

### Soft agar colony formation assay

Soft agar assays were performed on 6-well plates. The base layer of each well consisted of 2 ml (with a final concentration of 1×) medium and 0.6% low melting point agarose (Duchefa Biochemie, Netherland). Plates were chilled at 4 °C until solid. Next, 2 ml of growth agar layer was poured, consisting of 1 × 10^4^ cells suspended in 1× medium and 0.3% low-melting-point agarose; plates were again chilled at 4 °C until the growth layer congealed. Further 1 ml of 1× medium without agarose was added on top of the growth layer. Cells were incubated at 37 °C with 5% CO_2_ for approximately 14–21 days, and a total number of colonies were stained with 0.005% crystal violet (Sigma-Aldrich) and counted. Images were analyzed using an Olympus microscope (Olympus, Tokyo, Japan) and Image-Pro Plus 4.5 software (Media Cybernetics Inc., Rockville, MD, USA). Assays have been repeated a total of three times.

### Cell migration assay

In vitro cell migration assay was performed in a 24-well transwell plate with 8 μm polyethylene terephthalate membrane filters (BD Biosciences) separating the lower and upper culture chambers. Cells were grown until they reached sub-confluence (∼75–80%) and were serum-starved for 24 h. After detachment with trypsin, cells were washed with PBS and re-suspended in a serum-free medium, after which the cell suspension (2 × 10^4^ cells) was added to the upper chamber. Complete medium was added to the bottom wells of the chamber. The cells that had not migrated were removed from the upper surface of the filters using cotton swabs, and the cells that had migrated to the lower surface of the filters were fixed with 4% formaldehyde and stained with 0.2% crystal violet. Images of 3 random 10× fields were captured from each membrane, and the number of migratory cells was counted. The mean of triplicate assays for each experimental condition was used.

### Liquid chromatography–tandem mass spectrometry (LC–MS/MS) to identify the phosphorylation sites on Ephexin1

Flag-tagged Ephexin1 was transfected into HEK293T cells and 48 h after transfection, EGF (100 ng/ml) was added for 30 min. Thereafter, total cell lysates were immunoprecipitated with an anti-Flag antibody. Flag-Ephexin1 protein that was eluted from agarose beads using urea buffer (7 M urea, 2 M thiourea, 2% CHAPS) was diluted with 50 mM ammonium bicarbonate to ensure less than 1 M of urea content, reduced with 4 mM dithiothreitol (DTT) for 1 h at 37 °C, alkylated with 14 mM iodoacetamide (IAM) for 45 min at room temperature under dark condition, and excess IAM was quenched with excess DTT to provide a final concentration of 7 mM. Subsequently, the protein was digested with trypsin (Promega) at an enzyme content of 2% (w/w) for 16 h at 37 °C. To remove urea, the enzymatic peptides were cleaned up by ZipTip C18 (Millipore, USA). These peptides were dried by vacuum evaporation using a speed vac. Phosphopeptides of Ephexin1 from the tryptic peptides were enriched by Pierce^TM^ TiO_2_ phosphopeptide enrichment and clean-up kit (Thermo Scientific, USA) according to the manufacturer’s instructions. ABSciex TripleTOF^TM^ 5600 system that was coupled with Eksigent 1D + nano-LC system was used to identify phosphorylation sites of Ephexin1. The raw data was processed and searched for peptides using ProteinPliot^TM^ software (version 4.0) using Paragon^TM^ algorithm. Proteins were identified by searching the UniProtKB human database and filtered at ≥ 95% confidence cut off. Peptides for phosphorylated Ephexin1 were identified at 1% global FDR level.

### Human phospho-kinase array

H1299 cells (shControl or shEphexin1) were analyzed in the array panel of kinase phosphorylation profiles (Human Phospho-Kinase Array, ARY003B; R&D systems). Two hundred micrograms of cell lysates were incubated with the membrane. Thereafter, a cocktail of biotinylated detection antibias, streptavidin-HRP (1:2000), and chemiluminescent detection reagent was used for detecting phosphorylated kinase proteins. Membranes were then scanned and dot density was measured using image J software.

### In vitro GST-pulldown assay

Bacterially expressed GST-fusion DH/PH domain of Ephexin1 (residues 273-601) or GST alone was immobilized onto Glutathione Sepharose 4B beads (GE Healthcare) and incubated with bacterially expressed Hisx6-K-Ras fusion protein, overnight at 4 °C. The GST bead-bound complexes were then washed five times with GST lysis buffer (20 mM HEPES (pH 7.6), 150 mM NaCl, 5 mM MgCl_2_, 1% Triton X-100 and 5% Glycerol), and bound proteins were separated by SDS-PAGE and analyzed by western blotting using appropriate antibodies.

### In vitro small GTPase pulldown assay

The GTP-bound form of Ras was determined by using a GST-fusion protein of the Ras-binding domain (RBD) of Raf-1 (aa 1–149) as an activation-specific probe for endogenous Ras-GTP [34]. The recombinant GST-Pak1-PBD fusion protein, containing amino acids 56–141 of the CRIB-domain of Pak1B, was used as a probe for GTP-bound Rac1 and GTP-bound Cdc42 [35] and the recombinant Rhotekin–RBD fusion protein encoding the Rho-binding domain of Rhotekin, amino acids 7–89, was used as an activation-specific probe for RhoA-GTP [36, 37]. Twenty-four hours after transfection, cells were lysed in GST pulldown lysis buffer (25 mM Tris-HCl (pH 7.6), 150 mM NaCl, 5 mM MgCl_2_, 1% NP-40, 1 mM DTT, 5% Glycerol). Lysates were then clarified by centrifugation at 13,000 rpm for 10 min at 4 °C. GST-Rhotekin-RBD, GST-Pak1-PBD, and GST-Raf1-RBD with Glutathione Sepharose 4B beads were incubated with 1 mg of cell lysate in a final volume of 350 μl for 1 h at 4 °C. The beads were then washed three times with lysis buffer, and bound proteins were eluted in protein sample buffer and analyzed by 12% SDS-PAGE and western blotting.

### Tumor formation in nude mice

The mice used in this study were 6-week-old male BALB/c nude mice purchased from NARA Biotech (Seoul, South Korea). They were housed in our pathogen-free facility and handled in accordance with standard-use protocols and animal welfare regulations. HCT116, H1299, H460, and HEK293T cells were harvested and resuspended in PBS. Thereafter 2 × 10^6^ HCT116 cells, 2 × 10^6^ H1299 cells, 2 × 10^6^ H460 cells, and 1 × 10^6^ HEK293T cells were injected subcutaneously into the left and right flanks of BALB/c nude mice. The mice were sacrificed after 27–41 days, and the tumors were excised. Once the tumors became visible, the tumor size was measured every 3–5 days using micrometer calipers. Tumor volumes were calculated using the following formula: volume = 0.5*a* × *b*^2^, where *a* and *b* represent the larger and smaller tumor diameters, respectively. After approximately 4–6 weeks of injection, mice were humanely sacrificed and the primary tumors were excised, immediately weighed, fixed in 10% formalin solution (Sigma-Aldrich) and embedded in paraffin. All animal studies were reviewed and approved by the Institutional Animal Welfare and Use Committee.

### Immunostaining

Hematoxylin/eosin staining and immunohistochemistry (IHC) were performed on HCT116, H1299, and H460 cells and 8 μm thick sections of 10% formalin-fixed and paraffin-embedded mouse skin tissues, or tissue microarray (TMA) of colorectal and LC. TMA from colorectal and LC samples of different grades and adjacent normal tissues were purchased from Biomax (LC483; Rockville, MD, USA) and Super Bio Chips (CDA3 and CCA4; Seoul, South Korea). For IHC, heat-induced antigen retrieval was performed using 1× antigen retrieval buffer (pH 9.0) (Abcam) at 95 °C for 15 min. After quenching of endogenous peroxidase and blocking in 3% H_2_O_2_ solution, tissues were incubated with primary anti-Ephexin1 (PA5-52521, 1:100) (Thermo Scientific), anti-phospho-Ephexin1 Ser16/18 (8 μg/ml), anti-H3pS10 (#9701, 1:100), and anti-Ki67 (#9449, 1:100) antibodies (all from Cell Signaling) overnight at 4 °C, followed by incubation with HRP-conjugated secondary antibody (#711-035-152; Jackson ImmunoResearch, West Grove, PA, USA) for 1 h at room temperature and then incubated for 2 min in DAB substrate. The slides were then counterstained by dropping Harris’s hematoxylin. The intensity of staining was scored from 0 to 4, and the extent of staining was scored from 0 to 100%. The final quantitation score for each staining was obtained by multiplying the two scores. The slides were analyzed by two independent pathologists. For immunofluorescence, fluorophore-tagged secondary antibodies (#711-485-152; Jackson ImmunoResearch) were used and nuclei were counterstained with Hoechst 33258 (Sigma-Aldrich). Immunofluorescence was detected by a fluorescence microscope (Nikon, Japan).

### Statistical analysis

All data were analyzed using SPSS and presented with Graphpad Prism software 6.0. Differences between two independent groups were tested with Student’s *t*-test. Correlations between different parameters were analyzed using a two-way ANOVA test with Bonferroni’s post-test. Survival curves were plotted by the Kaplan–Meier method and compared by the log-rank test. For the nonparametric statistical test, the Mann–Whitney test was used. *P* value of less than 0.05 was considered statistically significant and *P* values were indicated by asterisks as followed: ^*^P < 0.05, ^**^P < 0.01, and ^***^P < 0.001, and n.s. = non-significant. Error bars represent standard deviations of three independent experiments. All experiments were performed in triplicate and repeated at least three times.

## Results

### Ephexin1 enhances proliferation in CRC and LC cells

To determine whether Ephexin1 expression is associated with tumorigenesis, we first analyzed Ephexin1 expression using RNA sequencing (RNA-seq) data from clinical samples included in (TCGA and GTEx database. When compared to the corresponding normal tissues, the levels of Ephexin1 in CRC (*n* = 639) and LC (*n* = 994) were more than 26-fold higher than that of normal sample tissues (Fig. 1A) and increased Ephexin1 expression in CRC and LC tissues correlated with poor overall survival (Fig. 1B). The IHC data showed that Ephexin1 protein levels were low or absent in normal samples but significantly elevated in the respective tumor samples (Fig. 1C). Consistent with the IHC and mRNA results, very low levels of Ephexin1 protein were detected in normal human lung and colon cells, whereas markedly higher levels were detected in most of the human CRC and LC cell lines, most of which carry the oncogenic Ras mutation (Fig. 1D and Supplementary Table S6).

We then generated an shRNA construct encoding an Ephexin1-targeting shRNA and used it to establish stable knockdown cells (Supplementary Fig. S1A). We found that the proliferative ability (Fig. 1E) and anchorage-independent growth (Fig. 1F) of the Ephexin1 knockdown cells were significantly inhibited compared to their control cells. Furthermore, there was a marked decrease in cell migration ability for all four depleted cell lines compared to control cells (Fig. 1G). Consistently, xenograft tumor growth in vivo as a result of injection of Ephexin1-depleted cells was significantly delayed compared to control cells (Fig. 1H, I and Supplementary Fig. S1B). Correspondingly, lack of Ephexin1 protein expression was associated with decreased tumor cell proliferation (Ki-67) (Supplementary Fig. S1C, D). Together, these results suggest that Ephexin1 may play an important role in the regulation of proliferation in CRC and LC.

### Ephexin1 promotes the MEK/ERK signaling pathway and contributes to increased expression of K-Ras target genes

**Fig. 2:**
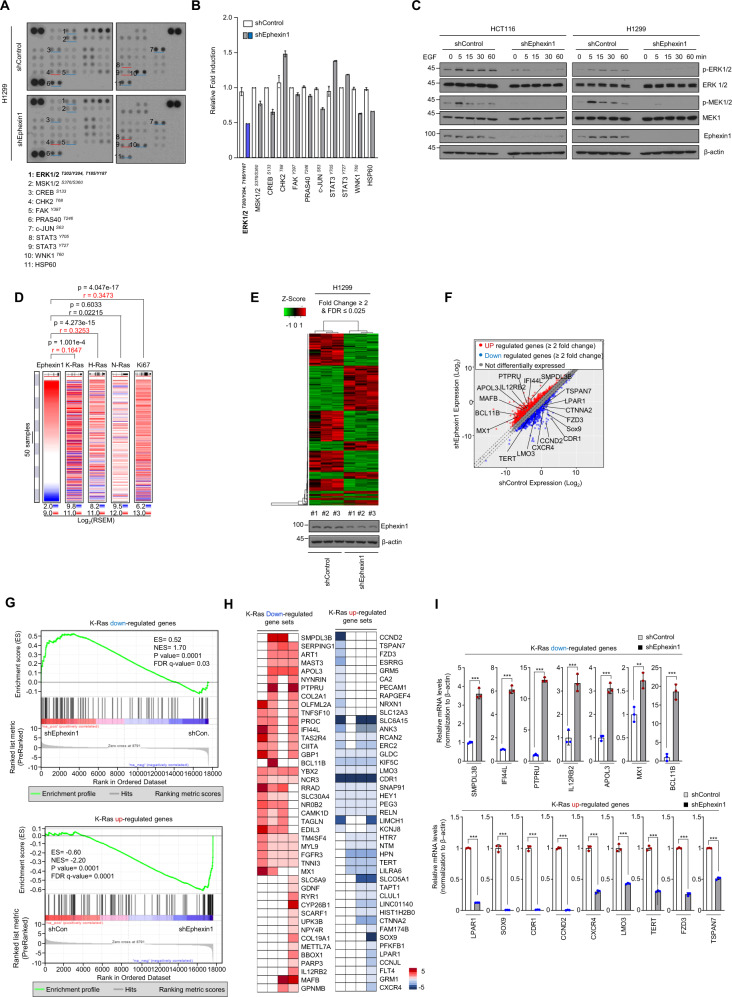
Ephexin1 enhances the MEK/ERK signaling pathway and regulates K-Ras-downstream target genes. **A** Whole-cell lysates from control or Ephexin1-depleted H1299 cells were incubated with human phosphokinase array membranes. Each pair of the most positive kinase dots is numbered with the identification of the corresponding kinase listed. **B** Levels of indicated protein kinases were quantified after normalized positive control. Error bars indicate mean ± SD of spots. **C** Western blot analysis to detect MEK/ERK activation in indicated cells treated with EGF for the indicated amounts of time after serum starvation. **D** The co-expression heat map of Ephexin1 with K-Ras, H-Ras, N-Ras, or Ki-67 in TCGA and GTEx (*n* = 5151) derived from the UCSC Xena browser. **E** Heat map showing the differential expressed genes induced by Ephexin1 shRNA in H1299 cells. Red and green indicate high and low mRNA expression levels, respectively. **F** Scatter plot shows the differently expressed genes (FDR ≤ 0.025 and fold difference ≥ 2) in Ephexin1-depleted H1299 cells compared with control cells. Compared to the control group (shControl), shEphexin1 downregulates 155 genes, whereas upregulates 227 genes expression levels. White circles highlight the position of the indicated K-Ras-downstream target genes. **G** Gene set enrichment analysis (GSEA) was used to identify K-Ras-downstream target genes differentially expressed between control and Ephexin1-depleted H1299 cells. **H** Heatmap of Ras-target genes downregulated and upregulated by Ephexin1 knockdown in H1299 cells. **I** RT-qPCR analysis of indicated genes. Values represent relative expression normalized to β-actin mRNA ± SD. ^**^P < 0.01; ^***^P < 0.001 compared with control. *P* values are for a two-tailed Student’s *t* test.

Human phosphokinase array assay revealed that the decrease in ERK phosphorylation by Ephexin1 knockdown was reduced (Fig. 2A, B). Western blot analysis was used to confirm that phosphorylation of ERK was reduced in Ephexin1-depleted cancer cells (Supplementary Fig. S2A–D). Reconstitution of Ephexin1 in Ephexin1-depleted H1299 cells partially rescued EGF-induced ERK phosphorylation (Supplementary Fig. S2E). Consistently, after serum starvation, HCT116 and H1299 cells (oncogenic Ras mutation) and HEK293T cells (wild-type Ras) with reduced Ephexin1 levels exhibited impaired EGF-induced phosphorylation of MEK and ERK (Fig. 2C and Supplementary Fig. S2F). To further analyze the effect of Ephexin1 on ERK phosphorylation and proliferation, we utilized the Ephexin1-sgRNA (Supplementary Fig. S3A, B). HCT116 cells with Ephexin1-sgRNA significantly inhibited ERK phosphorylation and cell growth compared with cells treated with negative control sgRNA (Supplementary Fig. S3C, D). These results suggest that Ephexin1 promotes Ras downstream MEK/ERK signaling pathway.

Given that Ephexin1 is one of the target genes in the Ras signaling pathway [28], we looked for a direct impact of Ras on the level of Ephexin1 expression using RNA-sequencing data from TCGA and GTEx samples (*n* = 5151) and found that expression of Ephexin1 and Ras, and expression of Ephexin1 and Ki67 are both significantly correlated (Fig. 2D). We then examined the effect of oncogenic Ras on Ephexin1 expression in HEK293T cells. The levels of Ephexin1 mRNA and protein both increased with HA-K-Ras^G12V^ expression and EGF treatment (Supplementary Fig. S4A–D). Ephexin1 expression was higher in cells expressing constitutively active K-Ras compared to wild-type (WT) K-Ras (Supplementary Fig. S4E, F). Similar results were also observed when HA-K-Ras^G12V^ was overexpressed in HCT116 and H1299 cells (Supplementary Fig. S4G, H). A protein turnover study revealed that when HA-K-Ras^G12V^ was overexpressed, the half-life of Ephexin1 increased (Supplementary Fig. S4I). These results suggest that oncogenic K-Ras upregulates Ephexin1 transcription and also increases the protein stability of Ephexin1.

To further verify a correlation between Ephexin1 and Ras, we performed RNA-seq analysis in Ephexin1 knockdown H1299 cells. Bioinformatic analysis revealed 382 differentially expressed genes (Fig. 2E). Importantly, genes normally upregulated by K-Ras were downregulated in Ephexin1 knockdown H1299 cells. And consistent with this trend, the genes normally downregulated by K-Ras were upregulated in Ephexin1 knockdown H1299 cells (Fig. 2F). GSEA and heatmaps confirmed this positive correlation between Ephexin1 expression and expression of K-Ras (Fig. 2G, H). Moreover, MEK (MAP2K1) target genes (MEK_UP.V1_UP: http://www.gsea-msigdb.org/gsea/msigdb/cards/MEK_UP.V1_UP.html) [38], but not PI3K-AKT target genes, were downregulated in Ephexin1 knockdown H1299 cells (Supplementary Fig. S5A). Similarly, when Ephexin1 was knocked down, ERK phosphorylation was decreased in a panel of human cancer cell lines carrying oncogenic Ras (HCT116, H1299, A549, and H460), whereas Akt phosphorylation was marginally affected (Supplementary Fig. S5B). The RNA-seq data have been deposited in the NCBI Gene Expression Omnibus (GEO) and are accessible through GEO series accession number GSE147809. The genes for which there was a particularly notable difference in Ephexin1-shRNA/H1299 cells compared to the control were confirmed using RT-qPCR (Fig. 2I). Together these findings suggest that K-Ras-induced upregulation of Ephexin1 expression contributes to the positive regulation of K-Ras-mediated downstream target genes.

### Ephexin1 interacts directly with oncogenic Ras and promotes the Raf activation

**Fig. 3:**
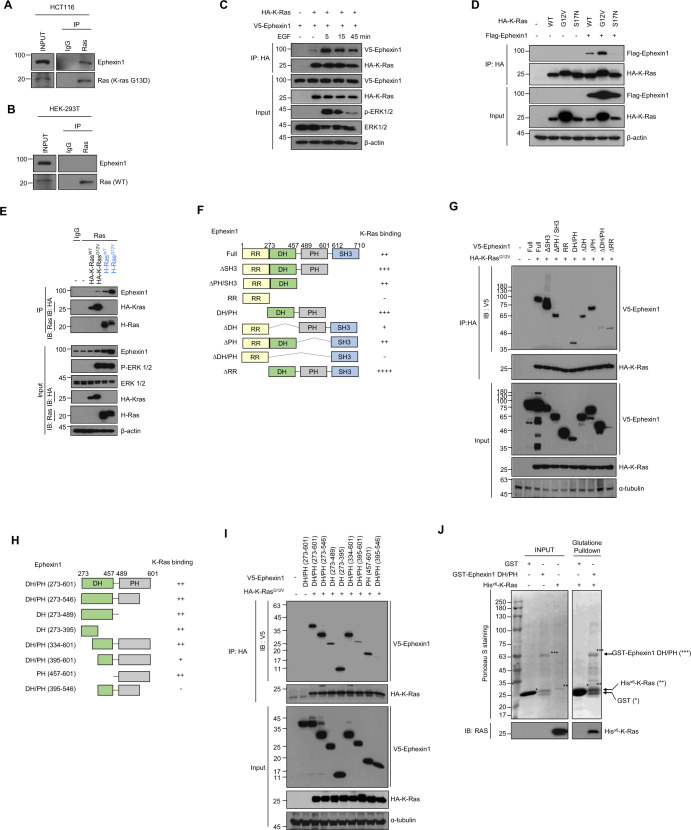
Ephexin1 interacts with oncogenic K-Ras via DH and PH domain. **A**, **B** HCT116 (**A**) and HEK293T (**B**) cells were immunoprecipitated with anti-Ras antibody and immunoblotted with anti-Ephexin1 and anti-Ras antibodies. **C** Co-immunoprecipitation (Co-IP) analysis of protein extracts from HEK293T cells cotransfected with HA-tagged K-Ras and V5-tagged Ephexin1, and treated with EGF (100 ng/ml) after 16 h serum starvation. IP was performed with a HA antibody, and the interaction of Ephexin1 to K-Ras was investigated by IB using an anti-V5 antibody. **D** Co-IP analysis of protein extracts from HEK293T cells cotransfected with HA-tagged K-Ras (WT, G12V, or S17N) and Flag-tagged Ephexin1. IP was performed with a HA antibody, and the interaction of Ephexin1 to K-Ras was investigated by western blot using an anti-Flag antibody. **E** HEK293T cells were transfected with HA-K-Ras^WT^, HA-K-Ras^G12V^, H-Ras^WT^, or H-Ras^G12V^, then cell lysates were immunoprecipitated with control IgG or anti-Ras antibody. The immunoprecipitates were then blotted with indicated antibodies. **F** Schematic representation of full length and a series of deletion mutants of Ephexin1. A summary of the degree to which each interacts with K-Ras is shown to the right. **G** Lysates from HEK293T cells cotransfected with V5-tagged full length or mutant Ephexin1 along with HA-tagged K-Ras^G12V^ were immunoprecipitated with anti-HA antibody and subjected to western blot analysis with V5 and HA antibodies. **H** Schematic representation of the DH/PH domain of Ephexin1 and a series of deletion mutants. A summary of the degree to which each interacts with K-Ras is shown to the right. **I** Lysates from HEK293T cells transiently expressing V5-tagged deletion mutants of Ephexin1 with HA-tagged K-Ras^G12V^ were immunoprecipitated with anti-HA antibody and then western blot with V5 and HA antibodies. **J** In vitro GST-pulldown assay of the binding of recombinant His-tagged K-Ras with GST or the GST-DH/PH domain of Ephexin1.

Given that Ephexin1 protein stability is increased by oncogenic Ras (Supplementary Fig. S4I), we predicted that there are direct interactions between the two proteins. To establish this, we immunoprecipitated Ras from HEK293T (WT Ras) and HCT116 (K-Ras mutation) cells. We found that endogenous Ras coprecipitated with endogenous Ephexin1 in HCT116 cells (Fig. 3A), whereas endogenous Ras from HEK293T cells did not coprecipitate with endogenous Ephexin1 (Fig. 3B). In addition, V5-Ephexin1 was observed in the HA-K-Ras-WT immunoprecipitate and this interaction was dramatically increased following treatment with EGF (Fig. 3C). Moreover, interactions were observed between Ephexin1 and both WT Ras (K-Ras and H-Ras) and oncogenic Ras but not with the K-Ras^S17N^ and H-Ras^S17N^, and the interaction of Ephexin1 with oncogenic K-Ras was much stronger than their WT (Fig. 3D, E and Supplementary Fig. S6A).

To identify the domain of Ephexin1 involved in binding to K-Ras, we generated V5-tagged constructs of eight different deletion mutants of Ephexin1 (Fig. 3F). HA-K-Ras^G12V^ co-immunoprecipitated with all of the deletion mutants except RR and ΔDH/PH (Fig. 3G). To further narrow the binding region, we created eight DH/PH domain mutants (Fig. 3H) and found that amino acids 273–395 and 547–601 are sufficient for HA-K-Ras^G12V^ binding (Fig. 3I). Immunoblotting of the GST pulldown samples revealed a direct interaction between the DH/PH domain of Ephexin1 and K-Ras (Fig. 3J).

Because Ephexin1 and oncogenic K-Ras interact directly, there is a possibility that Ephexin1 affects Ras activity. However, both Ephexin1 knockdown and overexpression did not affect Ras activity (Supplementary Fig. S6B, C). We thus assessed the phosphorylation status of Raf in the presence or absence of Ephexin1. Indeed, Ephexin1-depleted HEK293T cells had decreased levels of K-Ras^G12V^-induced phosphorylation of B- and C-Raf (Supplementary Fig. S6D). Consistently, Ephexin1-depleted H1299 and HCT116 cells exhibited impaired EGF-induced phosphorylation of B-Raf and C-Raf (Supplementary Fig. S6E). Furthermore, there was a significant reduction in phosphorylated B-Raf and C-Raf in the membrane fraction after EGF treatment in Ephexin1-depleted cells (Supplementary Fig. S6F). Moreover, upon knockdown of Ephexin1 in HEK293T cells, the binding of K-Ras^G12V^ to B-Raf and C-Raf was significantly reduced (Supplementary Fig. S6G–I). Taken together, these data suggest that Ephexin1 does not directly affect Ras activity but plays an important role in Raf activation by interaction with oncogenic Ras.

### Akt-mediated phosphorylation of serine 16/18 induces interactions between Ephexin1 and oncogenic Ras

**Fig. 4:**
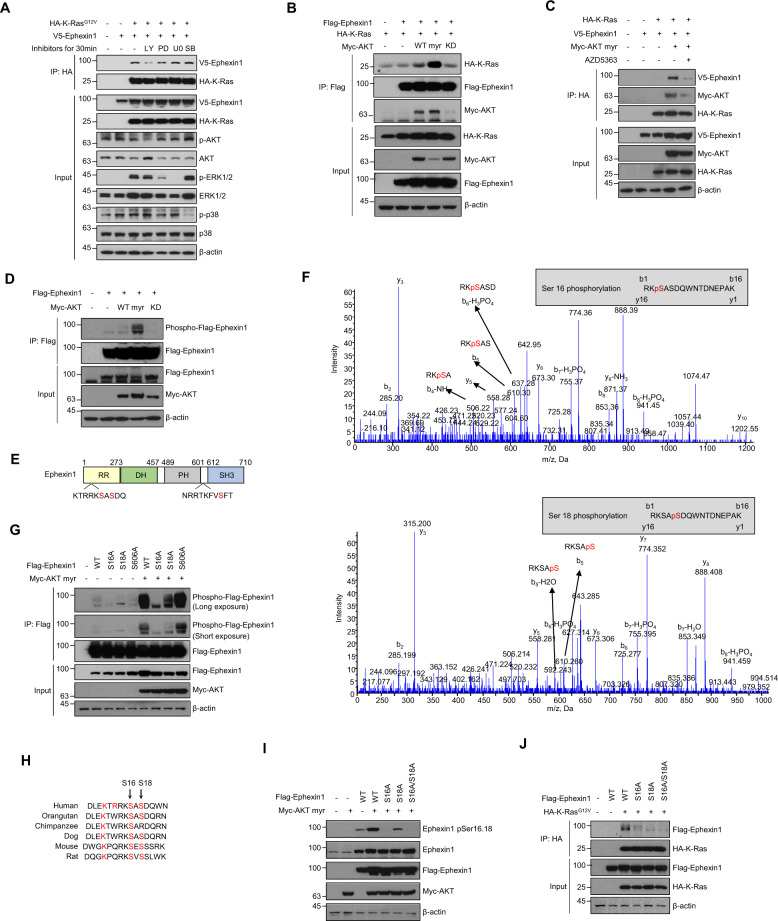
Akt phosphorylates Ephexin1 at Ser16 and Ser18. **A** Immunoprecipitation using an anti-HA antibody and western blot analysis using the indicated antibodies with HEK293T cells transfected with HA-K-Ras^G12V^ and V5-Ephexin1, and treated after 48 h with the following inhibitors for 30 min: PI3K inhibitor LY294002 (50 μM), MEK/ERK inhibitors PD98059 (50 μM), and U0126 (10 μM), and p38 inhibitor SB202190 (10 μM). **B** Co-IP analysis was conducted in HEK293T cells coexpressing Flag-tagged Ephexin1 and HA-tagged K-Ras along with Myc-tagged Akt (WT, myr, or KD). Cell lysates were immunoprecipitated with anti-Flag antibody, and the interaction of Ephexin1 to K-Ras was investigated by western blot using anti-HA antibody. **C** HEK293T cells were transfected with indicated plasmid, treated with 3 μM AZD5363 for 3 h, and subjected to immunoprecipitation followed by immunoblotting as indicated. **D** Flag-tagged Ephexin1 was coexpressed with Myc-tagged myr-Akt (WT, myr, or KD) in HEK293T cells. Cell lysates were immunoprecipitated with anti-Flag antibody, and the phosphorylation of Ephexin1 was analyzed by western blot with anti-phospho-Akt substrate antibody. **E** Schematic diagrams of the Ephexin1 protein domains with putative Akt phosphorylation sites indicated. **F** Determination of Akt catalyzed phosphorylation sites in Ephexin1 by mass spectrometry. Peptides contain Ser16 and Ser18 phosphorylation are shown at the top and bottom, respectively. **G** Flag-tagged WT or mutant Ephexin1 was coexpressed with Myc-tagged myr-Akt in HEK293T cells. Cell lysates were immunoprecipitated with an anti-Flag antibody, and the phosphorylation of Ephexin1 was analyzed by western blot with an anti-phospho-Akt substrate antibody. **H** Amino acid sequence alignment of a region of Ephexin1 with select conserved residues, including Ser16 and Ser18, is indicated in red. **I** Flag-tagged WT or mutant Ephexin1 was coexpressed with Myc-tagged myr-Akt in HEK293T cells. Phosphorylation of Ephexin1 at Ser16 and Ser18 was analyzed by western blot with anti-pSer16/18 Ephexin1 antibody. **J** Co-IP analysis was conducted in HEK293T cells cotransfected with HA-tagged K-Ras^G12V^ along with Flag-tagged WT or mutant Ephexin1. Cell lysates were immunoprecipitated with anti-HA antibody, and the interaction of K-Ras^G12V^ to Ephexin1 mutants was investigated by western blot using anti-Flag antibody.

To identify the signaling pathway that leads to the interaction of Ephexin1 with oncogenic K-Ras, we first assessed the effects of three specific types of inhibitors and found that the PI3K pathway inhibitor prevented binding of Ephexin1 to constitutively activated K-Ras (Fig. 4A). Coimmunoprecipitation analysis revealed that there was a stronger association between Ephexin1 and K-Ras when constitutively active Akt (myr-Akt), but not dominant-negative Akt (Akt-KD), was expressed (Fig. 4B). Consistently, the treatment of AKT inhibitor significantly inhibited the binding of K-Ras to Ephexin1 (Fig. 4C). Immunoblot analysis with anti-Akt substrate antibody showed enhanced Ephexin1 phosphorylation in anti-Flag-Ephexin1 immunoprecipitation complexes obtained from myr-Akt-expressing cells (Fig. 4D). Sequence analysis of Ephexin1, using the program *Scansite* (http://scansite.mit.edu), identified consensus Akt phosphorylation motifs at Ser16, Ser18, and Ser606 (Fig. 4E). LC–MS/MS analysis showed that two of these sites, Ser16 and Ser18, but not the third, Ser606, were phosphorylated in vivo (Fig. 4F). To validate these results, we generated site-specific alanine substitution mutations at Ser16, Ser18, and Ser606 and found significantly lower phosphorylation levels for the S16A and S18A mutants than for WT; whereas phosphorylation was unchanged in the S606A mutant (Fig. 4G). These two consecutive serine residues are conserved in mammalian species supporting the physiological significance of this phosphorylation event (Fig. 4H).

To further investigate the phosphorylation of Ephexin1 at Ser16 and Ser18 by Akt, we generated a polyclonal antibody that specifically binds pSer16/18 Ephexin1 and established the specificity of this antibody (Supplementary Fig. S7A). Using this antibody, we found that EGF and K-Ras^G12V^, which are known to promote activation of Akt, increased Ephexin1 phosphorylation, which was inhibited by Akt-KD (Supplementary Fig. S7B–F). Moreover, when either or both of the two serine residues are mutated to alanine, the level of Ephexin1 phosphorylation induced by myr-Akt was significantly reduced in HEK293T cells (Fig. 4I), confirming Ephexin1 phosphorylation at Ser16/18 in vivo. Notably, while WT Ephexin1 bound to HA-K-Ras^G12V^ robustly, none of the phosphomutants co-precipitated (Fig. 4J), Similarly, upon EGF treatment, WT Flag-Ephexin1 interacted strongly with WT K-Ras, but S16A/S18A mutant failed to do so (Supplementary Fig. S8A). Moreover, an Ephexin1 phosphomimetic mutant (S16D/S18D) is readily bound to K-Ras (Supplementary Fig. S8B). These results indicate that phosphorylation of these serine residues is important for the interaction between Ephexin1 and K-Ras.

### Phosphorylation of Ephexin1 at serine 16/18 promotes aberrant activation of the Ras/MAPK signaling pathway and cancer cell proliferation

Since Ephexin1 promotes Rho family GTPase activity [20], we investigated whether the Akt-mediated phosphorylation of Ephexin1 affects this activity. We found that the level of activity for each was comparable among HEK293T cells transfected with WT, S16A/S18A, or S16D/S18D Flag-Ephexin1, indicating that phosphorylation at Ser16/18 has no effect (Supplementary Fig. S9A).

**Fig. 5:**
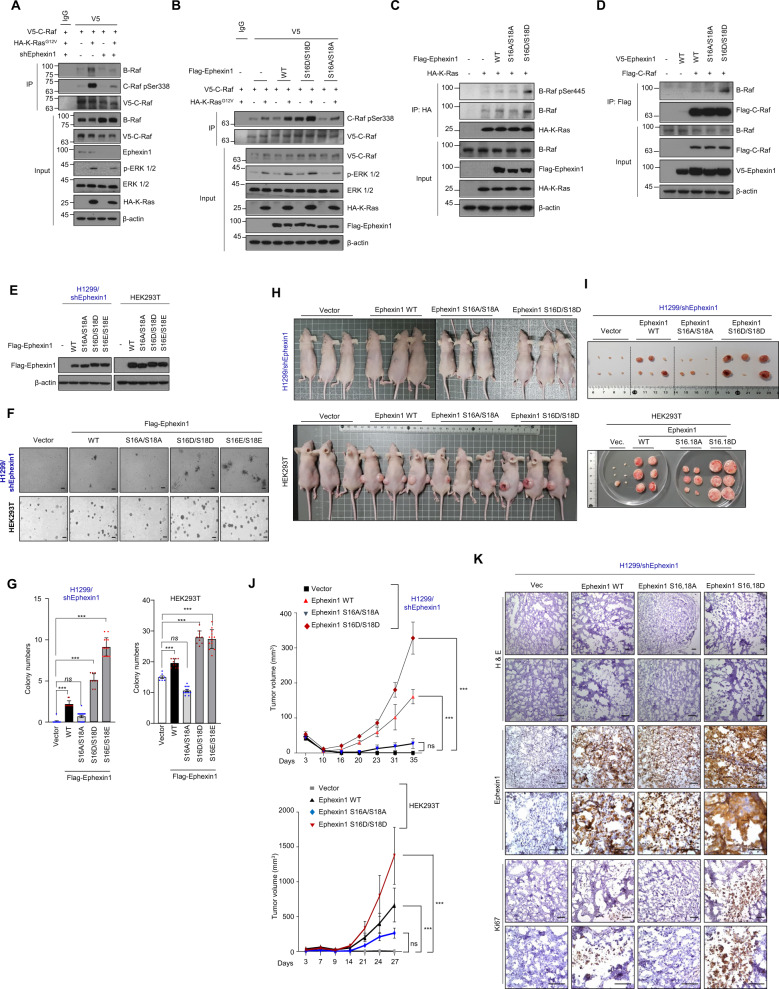
Phosphorylation of Ephexin1 at Ser16/18 promotes tumor cell growth and proliferation. **A** Lysates from control and Ephexin1-depleted HEK293T cells transfected with V5-tagged C-Raf along with or without HA-tagged K-Ras^G12V^ were immunoprecipitated with anti-V5 antibody and subjected to western blot analysis with indicated antibodies. **B** Co-IP analysis was conducted in HEK293T cells cotransfected with V5-C-Raf and Flag-Ephexin1 (WT, S16D/S18D, or S16A/S18A) with or without HA-K-Ras^G12V^. Cell lysates were immunoprecipitated with anti-V5 antibody and subjected to western blot analysis with indicated antibodies. **C** Co-IP analysis was conducted in HEK293T cells cotransfected with WT HA-K-Ras and Flag-Ephexin1 (WT, S16A/S18A, or S16D/S18D). Cell lysates were immunoprecipitated with anti-HA antibody and subjected to western blot analysis with indicated antibodies. **D** Co-IP analysis was conducted in HEK293T cells cotransfected with Flag-C-Raf and V5-Ephexin1 (WT, S16A/S18A, or S16D/S18D). Cell lysates were immunoprecipitated with anti-Flag antibody and subjected to western blot analysis with indicated antibodies. **E** Western blot analysis for Ephexin1 expression in indicated Ephexin1-depleted H1299 and HEK293T cells. **F** Anchorage-independent growth assessed by soft agar assay in Ephexin1-depleted H1299 cells transfected with Flag-tagged WT or mutant Ephexin1 (top) or in HEK293T cells transfected Flag-tagged WT or mutant Ephexin1 (bottom). Representative images are shown. Scale bar = 100 μm. **G** Statistical analysis between control and Ephexin1 (control or mutant) transfected cells in soft agar assay. Data are shown as mean ± SD. ns not significant, ^***^P < 0.001, two-tailed Student’s *t* test. **H** Ephexin1-depleted H1299 and HEK293T cells transfected WT or mutant Ephexin1 were inoculated subcutaneously in BALB/c nude mice. **I** Tumors derived from the indicated cells at endpoint (*n* = 6 mice per group). **J** Growth curves of tumors derived from the indicated cells in mice. Data are shown as mean ± SD. ns not significant, ^***^P < 0.001, two-way ANOVA. **K** H&E, Ki67, and Ephexin1 IHC stating analyses of H1299 xenograft tumors. Scale bar = 100 μm.

Next, we looked at whether pSer16/18 Ephexin1 positively regulates the Raf/MEK/ERK signaling. The HA-K-Ras^G12V^-mediated enhanced interaction between C-Raf and B-Raf was significantly reduced in cells lacking Ephexin1 but increased in Ephexin1-overexpressing cells (Fig. 5a and Supplementary Fig. S9B). Moreover, expression of S16D/S18D Ephexin1 but not the S16A/S18A mutant in HEK293T cells led to increased basal and HA-K-Ras^G12V^-mediated C-Raf phosphorylation (Fig. 5B). Notably, the interaction between WT K-Ras and phosphorylated B-Raf was significantly increased in S16D/S18D Ephexin1-overexpressing cells (Fig. 5C). Furthermore, the expression of S16D/S18D Ephexin1 led to increased interaction between C-Raf and B-Raf (Fig. 5D). These data provide strong evidence that phosphorylation Ser 16/18 of Ephexin1 causes aberrant activation of the Ras/Raf/MEK/ERK signaling pathway.

We then investigated whether phosphorylation of Ephexin1 by Akt plays a role in tumorigenesis. Reconstitution of WT Ephexin1 in Ephexin1-depleted H1299 cells was sufficient to increase anchorage-independent growth (Fig. 5E–G) and xenograft tumor in nude mice growth (Fig. 5H–J), but a more dramatic increase was seen in the presence of either the S16D/S18D or S16E/S18E mutant. On the other hand, Ephexin1-depleted H1299 cells reconstituted with S16A/S18A Ephexin1 remained unchanged. Consistently, reconstitution of S16A/S18A Ephexin1 showed a lower cell proliferation as determined by Ki-67 IHC staining (Fig. 5K). Similar results were also obtained in HEK293T cells transfected with either WT, S16D/S18D, S16E/S18E, or S16A/S18A Ephexin1 (Fig. 5E–J). Taken together, these findings support the conclusion that Akt-mediated phosphorylation of Ephexin1 S16/S18 stimulates cancer cell proliferation in vitro and in vivo.

### Phosphorylated Ephexin1 is clinically relevant in both CRC and LC

**Fig. 6:**
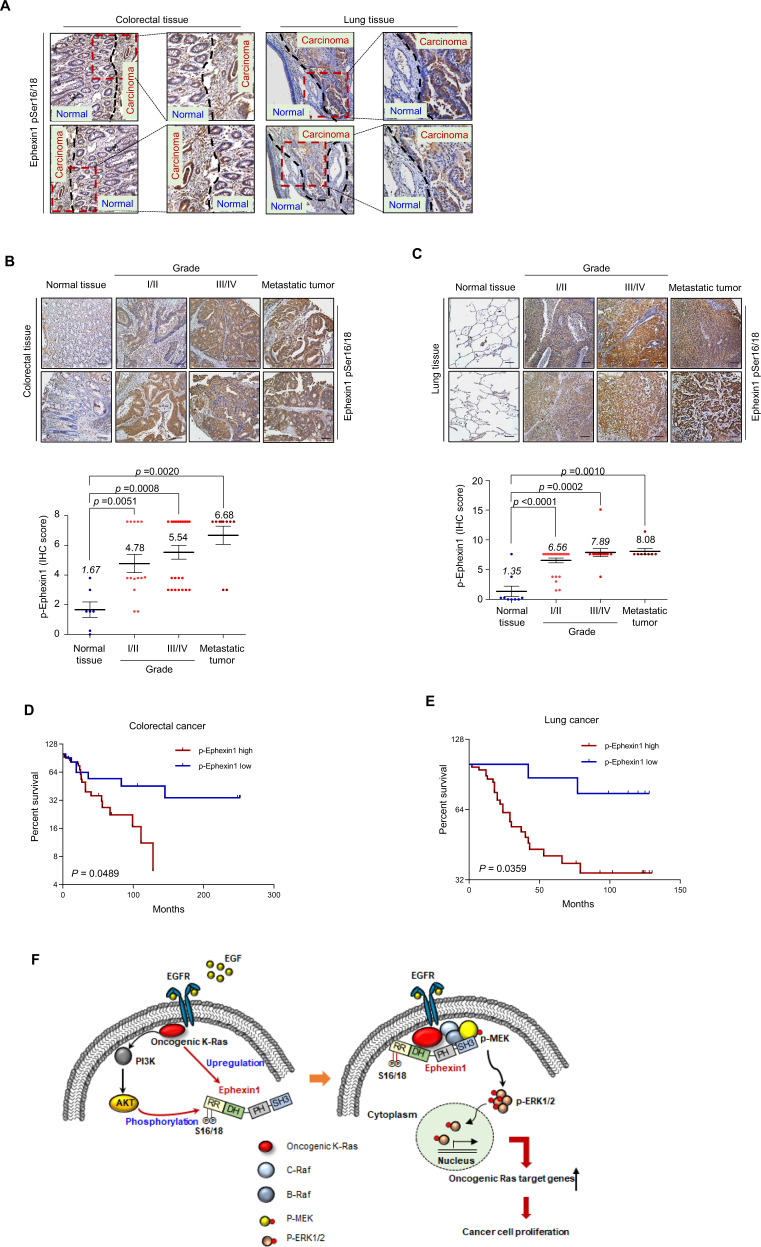
Ephexin1 is phosphorylated at Ser16/18 in human CRC and LC tissues and is associated with poor prognoses. **A** Representative image of pSer16/18 Ephexin1 immunoreactivity in normal and cancerous colorectal and lung tissues (separated by dashed lines). **B**, **C** IHC staining was performed to evaluate pSer16/18 Ephexin1 expression in normal, grade I/II, grade III/IV, and metastatic CRC (**B**) and LC (**C**) tissues and their corresponding normal tissues. Hematoxylin is the counterstain. Scale bar = 100 μm. Expression scores of pSer16/18 Ephexin1 in CRC tissue microarray (TMA) that was containing 9 cases of normal mucosa tissues, 14 cases grade I/II, 26 cases grade III/IV, and 10 cases of metastatic tumor tissues. Expression scores of pSer16/18 Ephexin1 in LC TMA that was containing 9 cases of normal mucosa tissues, 27 cases grade I/II, 13 cases grade III/IV, and 10 cases of metastatic tumor tissues. Data are shown as mean ± SD, *P* values are for a Mann–Whitney test. **D** A Kaplan–Meier graph representing overall survival rates for patients with pSer16/18 Ephexin1-overexpressing CRC. High pSer16/18 Ephexin1 expression, *n* = 33; low pSer16/18 Ephexin1 expression, *n* = 12. *P* values are for a log-rank test. **E** As in (**D**) but for LC. High pSer16/18 Ephexin1 expression, *n* = 42; low pSer16/18 Ephexin1 expression, *n* = 8. *P* values are for a log-rank test. **F** Schematic representation of a model for the major mechanism of the tumorigenic effect of Akt-mediated Ephexin1 phosphorylation on CRC and LC.

To investigate the clinical significance of pSer16/18 Ephexin1 in CRC and LC specimens, we examined pSer16/18 Ephexin1 expression levels by analyzing TMA on colorectal and lung tissues consisting of normal cells, carcinomas of different grades, and metastatic tumors. The expression of pSer16/18 Ephexin1 was markedly higher in CRC and LC than the corresponding normal tissues (Fig. 6A) and increased progressively and significantly with increasing tumor cell grade and metastatic tumors (Fig. 6B, C). The phosphorylated Ephexin1 antigenic peptide blocked staining by pSer16/18 Ephexin1 antibody, demonstrating that the staining of pSer16/18 Ephexin1 was specific (Supplementary Fig. S10). Kaplan–Meier survival analysis showed that a patient with high pSer16/18 Ephexin1 expression in CRC and LC tumors had markedly shorter survival than a patient with low pSer16/18 Ephexin1 expression (Fig. 6D, E). These results demonstrate the clinical importance of pSer16/18-Ephexin1 in human CRC and LC and support the rationale of targeting Ephexin1 in the development of anticancer drugs.

## Discussion

In the present study, we identified Ephexin1 as a newly identified positive regulator of oncogenic Ras-mediated cancer proliferation. Using TCGA data mining, TMA analysis, cancer cell systems, and xenograft models, we conclude that Ephexin1 plays a significant role in the functional and clinical significance of both CRC and LC, especially those triggered by a K-Ras mutation. These findings are highly indicative of a causal relationship between Ephexin1 expression and Ras mutation-mediated tumor-promoting pathway.

Secondly, a notable result here is that in its emerging role in Ras signaling, Ephexin1 binds directly to oncogenic Ras through the DH/PH domain. Mechanistically, we identify Ephexin1 as a novel substrate of Akt, which phosphorylates the Ser16 and Ser18 residues in the N-terminal helix of Ephexin1. The DH domain of Ephexin1 interacts with the N-terminal helix to exclude and prevent the activation of Rho GTPase [39]. Src- and EphA4-dependent phosphorylation of the N-terminal helix of Ephexin1 prevents the association of the autoinhibitory helix (N-terminal domain of Ephexin1) with the DH domain to promote the activation of Rho GTPase activity [23, 27]. Phosphorylation or deletion of the blocking N-terminal helix relieves this autoinhibition. Therefore, we speculate that Akt-dependent phosphorylation at the Ser16 and Ser18 residues of Ephexin1 may contribute to the conformational change of N-terminal helix on the DH and PH domains, which, in turn, triggers an interaction between Ephexin1 and oncogenic K-Ras through a mechanism that will require further investigation.

Thirdly, we found that Akt-mediated phosphorylation at Ser16 and Ser18 of Ephexin1 promotes interaction between Ephexin1 and K-Ras and triggers tumorigenesis. In noncancerous human embryonic kidney 293T cells, overexpression of the phosphomimetic mutants (S16D/S18D or S16E/S18E) but not the phosphomutant (S16A/S18A) in Ephexin1 conferred anchorage-independent growth as well as xenograft growth, suggestive of a contributing role for pS16/S18 Ephexin1 in the transformation process. In particular, in LC H1299 cell lines, Ephexin1 binding to oncogenic Ras is necessary for Ephexin1 tumorigenic activity, as S16A/S18A Ephexin1 did not bind to K-Ras and did not promote anchorage-independent growth and tumor formation in mice, while S16D/S18D Ephexin1, which binds to Ras, was capable of enhancing both anchorage-independent growth in vitro and tumor growth in vivo. Expression levels of pS16/S18 Ephexin1 were associated with increasingly malignant grades of CRC and LC and metastatic cancer, and high pS16/S18 Ephexin1 expression correlated with poor prognosis in CRC and LC patients. Ephexin1 promotes Rho GTPase activity, which is a known component of the Ras transformation program and is involved in a positive feedback loop for the MAPK pathway [40–43]. However, our data suggest that pSer16/S18 Ephexin1 increased MAPK signaling independent of its Rho GTPase activity, raising the possibility that the oncogenic potential of Ephexin1 may be mediated through its capacity to bind to K-Ras and subsequent activation of the MAPK pathway. Nevertheless, we could not exclude the possibility that the Rho GTPase activity of Ephexin1 [20, 21] may be involved in K-Ras-driven tumorigenicity.

The development of therapeutics that directly target mutant K-Ras has been hampered by a plethora of unfavorable factors, including an incomplete understanding of signaling transduction, feedback loops, redundancy and tumor heterogeneity, and difficulty in developing small-molecule inhibitors against Ras oncoproteins [44]. Rather, the identification of proteins that are both differentially expressed between normal and cancer cells and that participate in the activation of MEK/ERK is a better route for identifying important targets to selectively kill tumor cells. Ephexin1 may be such a target as it is rarely expressed in normal non-neuronal cells, is present at significantly elevated levels in CRC and LC cells and tissues, and is directly involved in Ras-mediated cancer cell proliferation and tumorigenicity. Moreover, because the phosphorylation of Ephexin1 at Ser16/18 by Akt, which is required for binding to K-Ras, is the key behind its tumor-promoting effects, it is reasonable to speculate that suppression of Ephexin1 phosphorylation will eliminate the tumorigenic effect in Ras-driven cancers. Recent data suggest that a peptide corresponding to the ERK1/ERK2-binding domain of the scaffold protein IQGAP1 inhibits Ras- and Raf-driven tumorigenesis and acts as a systemically deliverable therapeutic [45]. Therefore, the use of peptides or chemicals to inhibit the phosphorylation of Ephexin1 Ser16/18 may provide a novel approach to target K-Ras by selectively blocking the specific interaction between K-Ras and Ephexin1.

In summary, Ephexin1 is upregulated by an oncogenic K-Ras mutant, and when phosphorylated at S16 and S18 by Akt, interacts directly with K-Ras, thereby activating the Raf/MEK/ERK signaling pathway and modulating the expression of K-Ras-mediated downstream target genes, which finally leads to increased tumor cell growth and proliferation (Fig. 6F). Inhibition of Ephexin1 phosphorylation is a promising way in which to block Ras oncogenic activity without affecting Ras–MAPK signaling functions in normal non-cancerous cells.

## Supplementary information

Supplementary Figures and Tables

## Data Availability

The RNA-seq data were deposited in the NCBI Gene Expression Omnibus (GEO) and are accessible through GEO Series accession number GSE147809.
