# Supplementary material for: Akt-mediated Ephexin1–Ras interaction promotes oncogenic Ras signaling and colorectal and lung cancer cell proliferation
Source: Cell Death Dis. 2021 Oct 28;12(11):1013. doi: 10.1038/s41419-021-04332-0 (PMC8553951; doi:10.1038/s41419-021-04332-0)
Supplement: Supplementary file 1 — Supplementary Figures and Tables [file 41419_2021_4332_MOESM1_ESM.docx]

**
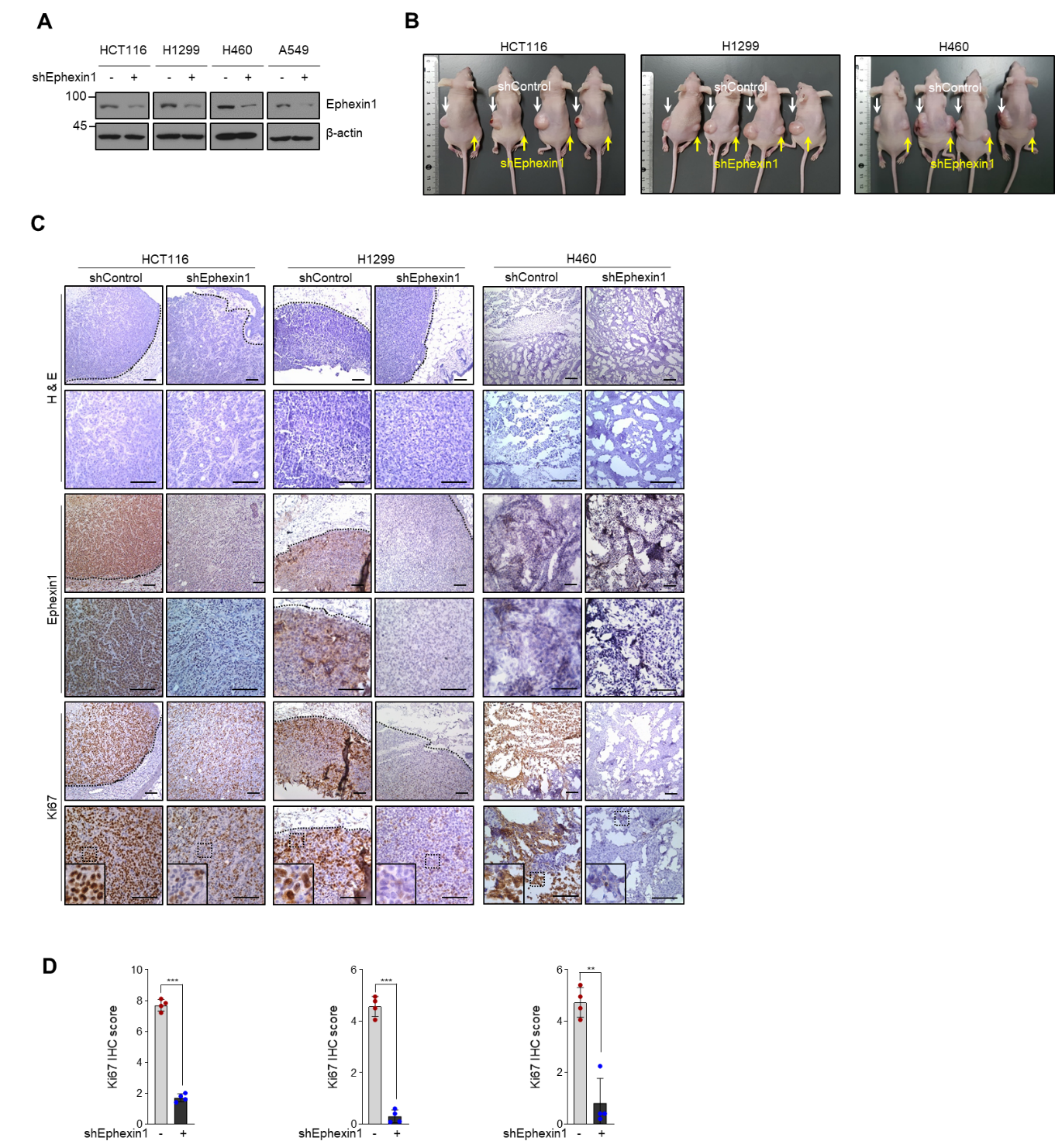
**

**Supplementary Fig. S1 Effect of Ephexin1 siRNA on the Ephexin1 expression and cancer cell proliferation. a** Control and Ephexin1 shRNA were stably transfected into HCT116, H1299, H460, and A549 cells. Ephexin1 protein expression was examined by western blot. **b** Control and Ephexin1-depleted HCT116, H1299 and H460 cells were inoculated subcutaneously in BALB/C nude mice (n = 4). **c** H&E, Ki67, and Ephexin1 IHC stating analyses of HCT116, H1299 and H460 xenograft tumors. Scale bar = 100 μm. **d** Quantification of the proliferation index (Ki67 staining) in HCT116, H1299, and H460 xenograft tumors. Data are shown as mean ± SD. ***P* < 0.01, and ****P* < 0.001, two-tailed Student’s *t*-test.


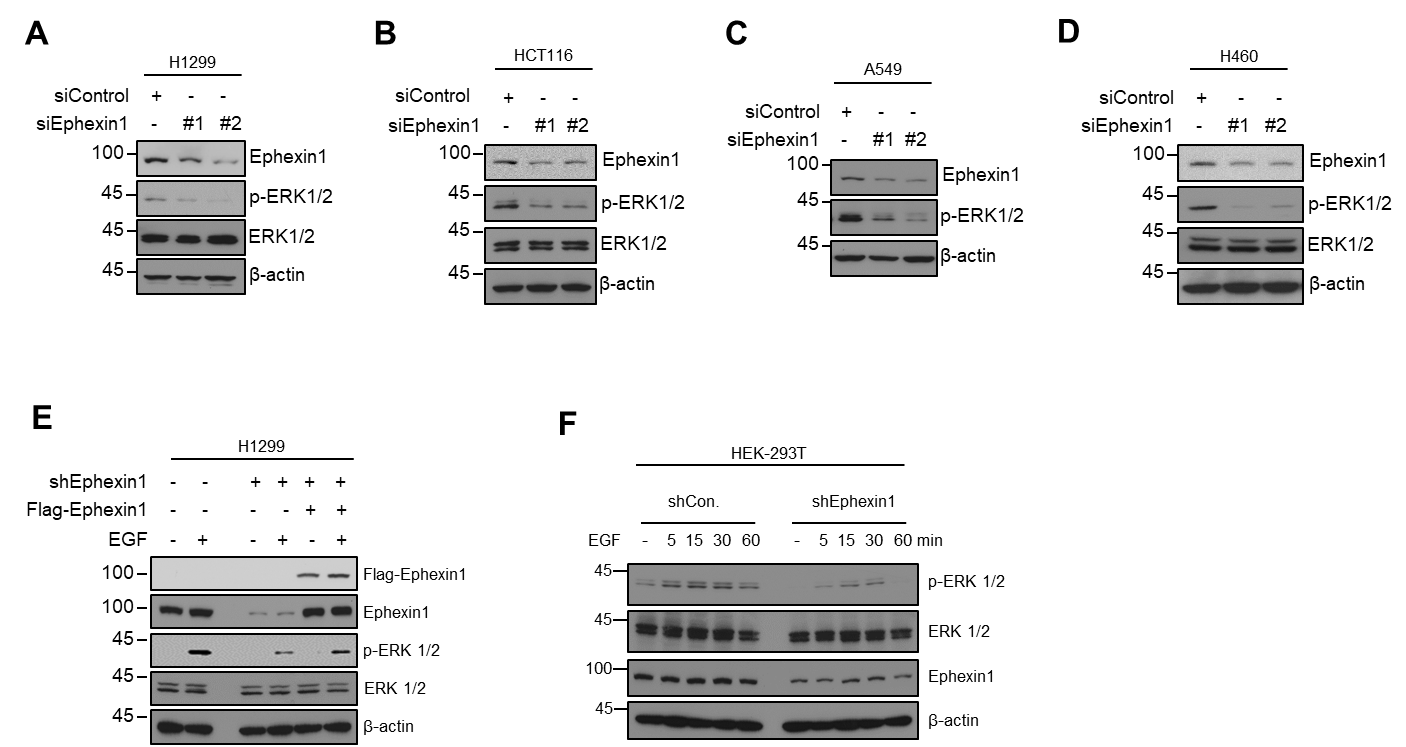


**Supplementary Fig. S2 Ephexin1 promotes ERK phosphorylation. a-d** H1299 (**a**), HCT116 (**b**), A549 (**c**), and H460 (**d**) cells were transiently transfected either control siRNA or two different Ephexin1 siRNAs, and the levels of endogenous Ephexin1, p-ERK1/2, and ERK1/2 were analyzed by western blotting. **e** Control and Ephexin1-depleted H1299 cells transfected with or without siRNA resistant Flag-Ephexin1 were treated with or without EGF (100 ng/ml) for 15 min. The p-ERK1/2, ERK1/2, and Flag-Ephexin1 were analyzed by western blotting using the indicated antibodies. **f** Control and Ephexin1-depleted HEK293T cells were treated with EGF (100 ng/ml) for the indicated amounts of time after 16 h of serum starvation. Cell lysates were western blot with indicated antibodies


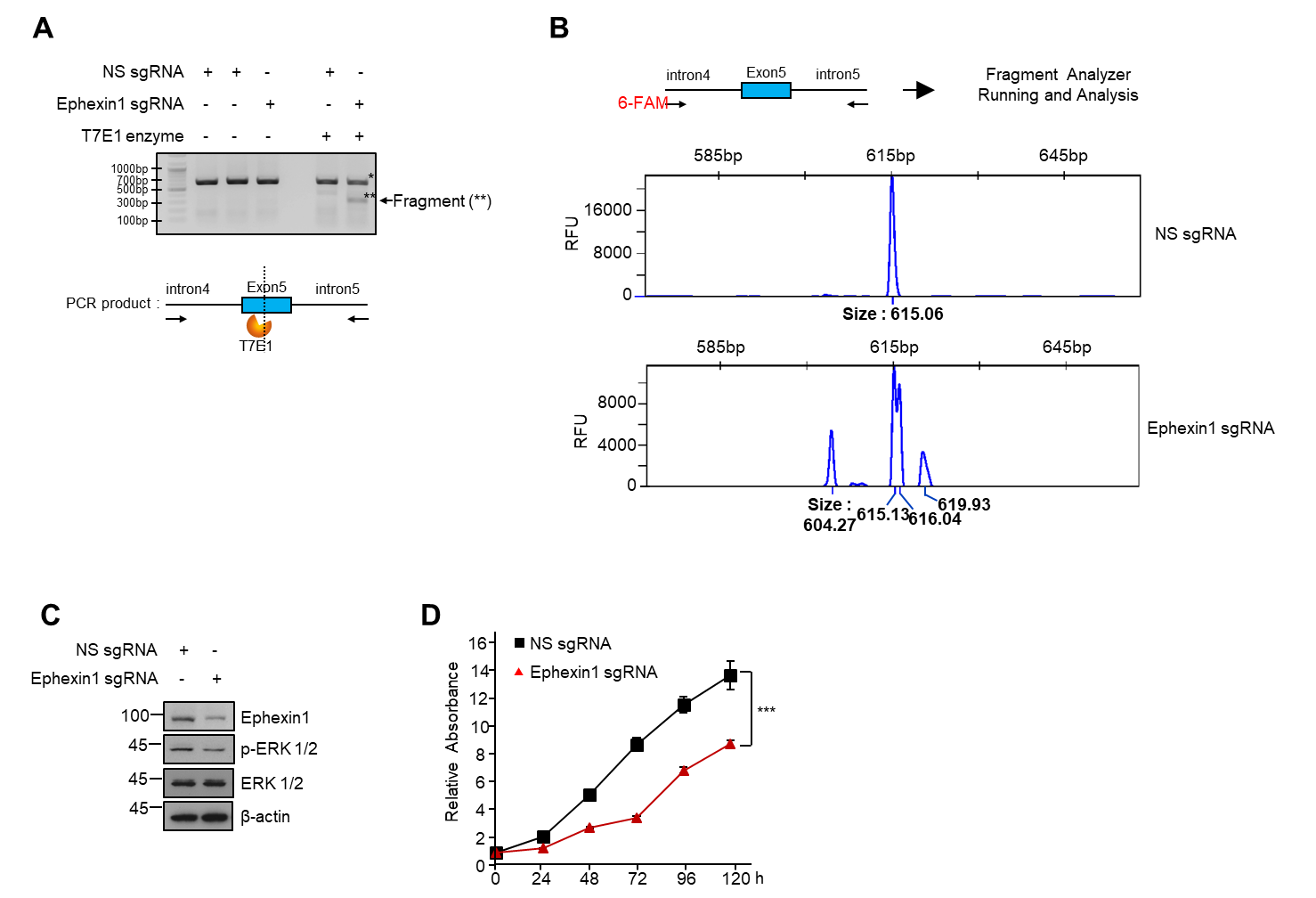


**Supplementary Fig. S3 Knockdown of Ephexin1 with the CRISPR/Cas9 system abrogates the ERK phosphorylation and proliferation in HCT116 cells. a** T7E1 analysis of CRISPR/Cas9-mediated Ephexin1 gene editing in HCT116 cells. Isolated gDNA was subjected to PCR amplification using Ephexin1 gene target site-specific primer set. Assay substrates are indicated by an asterisk (*), T7E1 cleavage products are indicated by double-asterisk (**). **b** Fragment analysis to evaluate gRNA. Forward primers labeled with 6-FAM were used for PCR amplification for fragment analysis. 615bp is the peak of the wild-type Ephexin1 clone, and 604bp, 616bp, and 620bp are the peaks of the Ephexin1 mutant clones. **c** HCT116 cells were transfected either Ephexin1 sgRNA or negative control sgRNA, and the levels of endogenous Ephexin1, p-ERK1/2, and ERK1/2 were analyzed by western blotting. **d** The Effect of Ephexin1 sgRNA on cell proliferation by MTT assay in HCT116 cells. Data are shown as mean ± SD. ***P < 0.001, two-way ANOVA.


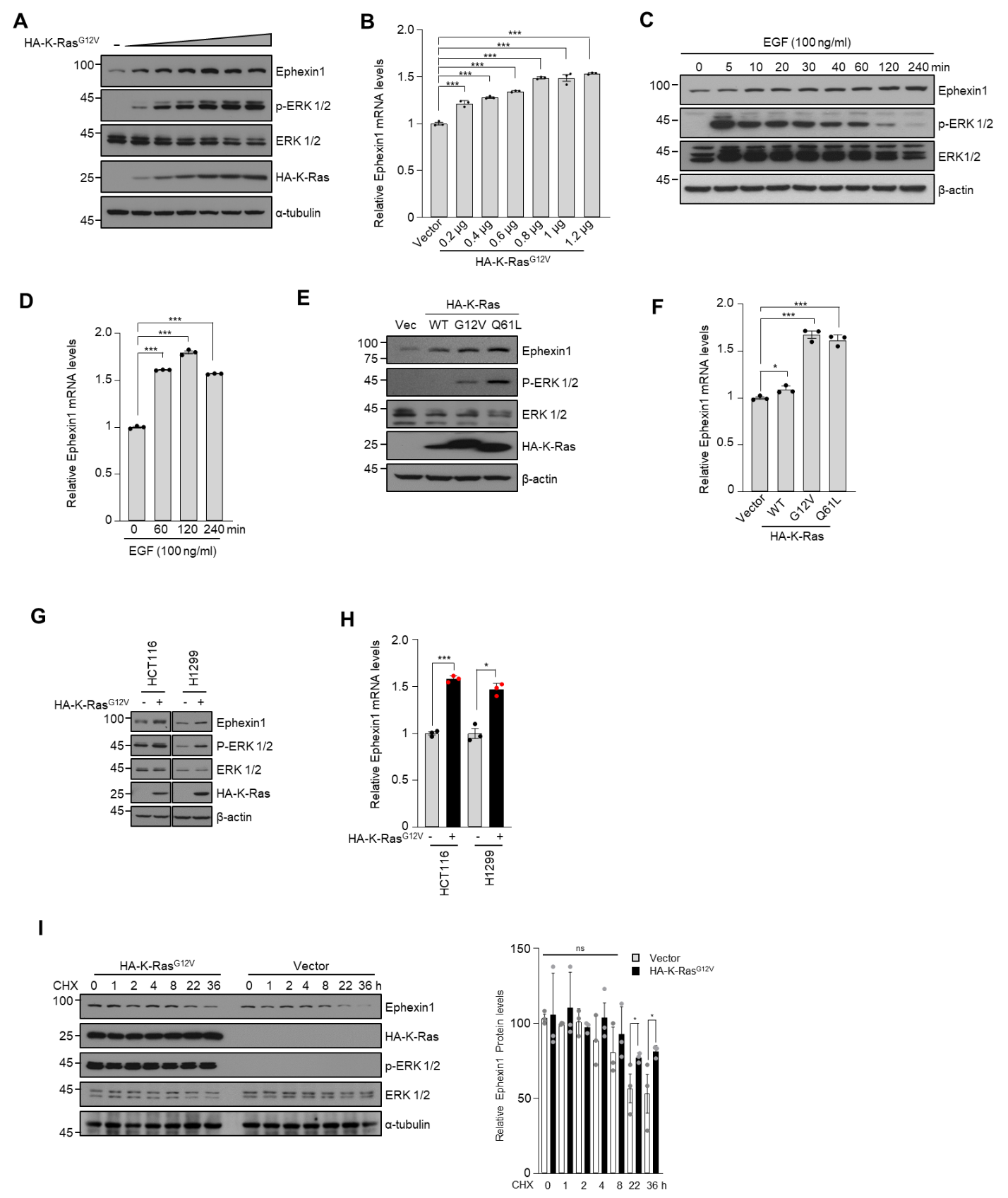


**Supplementary Fig. S4 Oncogenic K-Ras upregulates Ephexin1 expression through increasing its transcript and its protein stability. a,b** The levels of Ephexin1 protein (**a**) and mRNA (**b**) in HEK293T cells transfected with either control vector or increasing amounts of HA-tagged K-RasG12V. Data are shown as mean ± SD. ***P < 0.001, two-tailed Student’s t-test. **c** Western blot analysis with the indicated antibodies to measure the expression of Ephexin1, p-ERK1/2, and ERK1/2 in HEK293T cells treated with EGF (100 ng/ml) for the indicated amounts of time. **d** Ephexin1 mRNA expression levels from three independent experiments performed as in **c**. Data are shown as mean ± SD. ***P < 0.001, two-tailed Student’s t-test. **e** Western blot analysis using the indicated antibodies to measure the levels of Ephexin1, p-ERK1/2 and ERK1/2 48 h after transfection of HEK293T cells with either control vector or HA-tagged WT or mutant K-Ras (G12V or Q61L) plasmids. **f** Ephexin1 mRNA expression levels from three independent experiments performed as in j. Data are shown as mean ± SD. *P < 0.05, ***P < 0.001, two tailed Student’s t-test. **g, h** The levels of Ephexin1 protein (**g**) and mRNA (**h**) in HCT116 and H1299 cells transfected with control or HA-K-RasG12V expression plasmid. Data are shown as mean ± SD. *P < 0.05 and ***P < 0.001, two-tailed Student’s t-test. **i** Ephexin1 protein stability 48 h after transfection of cells with either control vector or HA-tagged K-RasG12V expression vector following cycloheximide treatment (10 μg/ml) for the times indicated. Data are shown as mean ± SD. ns, not significant, *P < 0.05, two-tailed Student’s t-test.


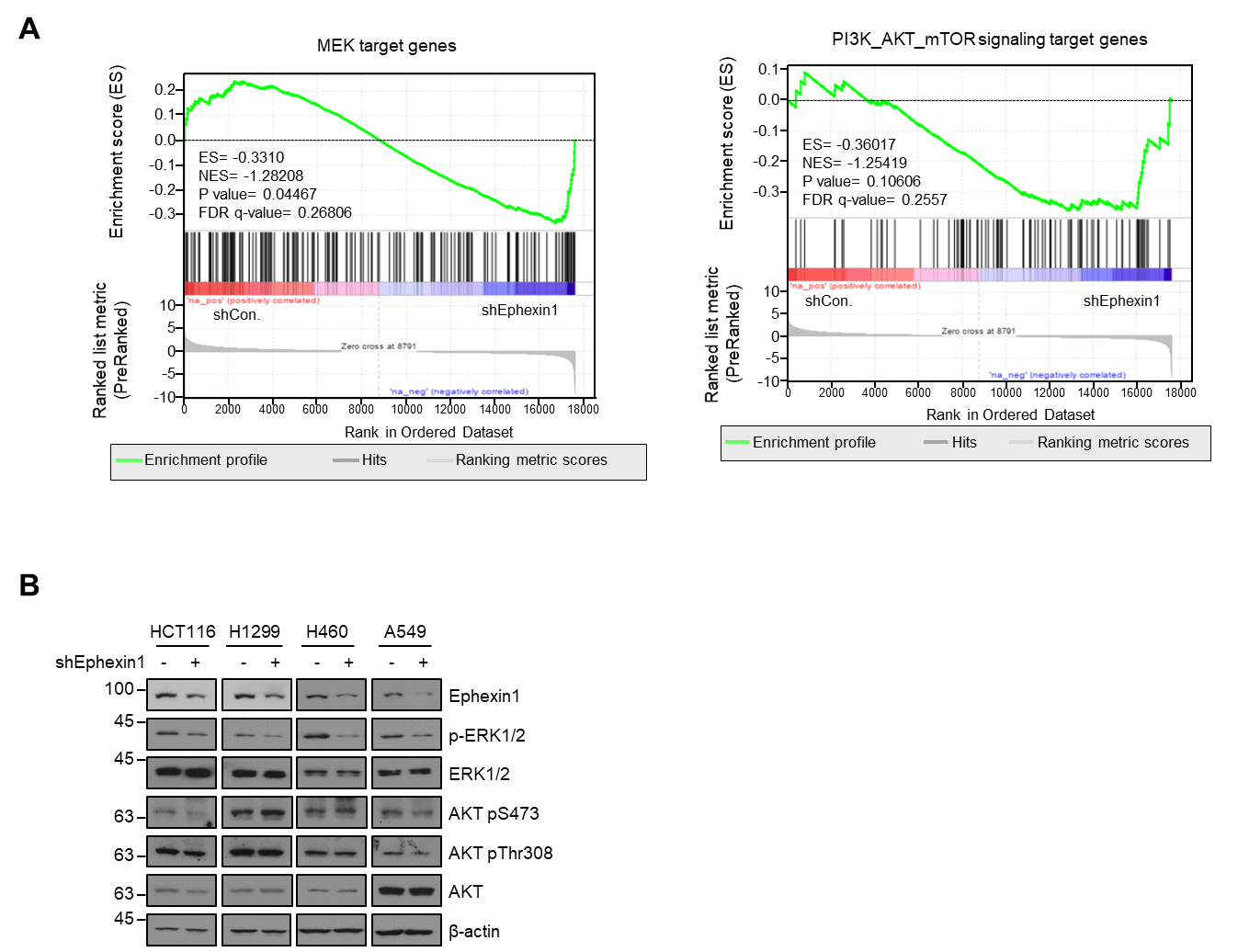


**Supplementary Fig. S5 Ephexin1 enhances the MEK/ERK signaling pathway.** **a** Gene set enrichment analysis (GSEA) was used to identify MEK (MAP2K1) target genes (MEK_UP.V1_UP: <http://www.gsea-msigdb.org/gsea/msigdb/cards/MEK_UP.V1_UP.html>) and PI3K / AKT / mTOR signaling target genes (HALLMARK_PI3K_AKT_MTOR_SIGNALING: <https://www.gsea-msigdb.org/gsea/msigdb/cards/HALLMARK_PI3K_AKT_MTOR_SIGNALING.html>) and differentially expressed between control and Ephexin1-depleted H1299 cells. **b** Western blot analysis of ERK and AKT activation in HCT116, H1299, H460, and A549 cells stably expressing either control or Ephexin1-targeting shRNA.

**
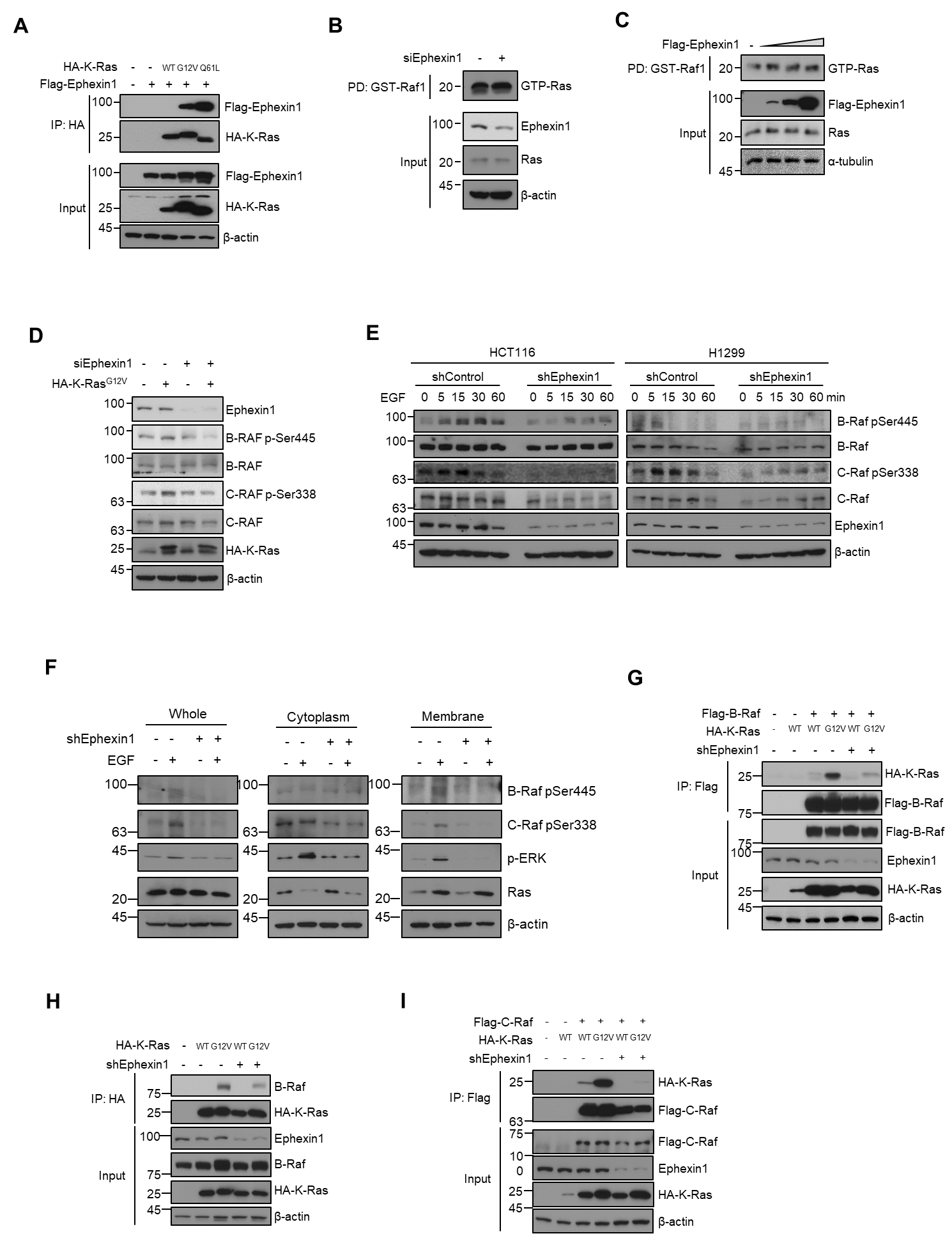
**

**Supplementary Fig. S6** **Ephexin1 does not contribute to the activation of Ras.** **a** Co-immunoprecipitation analysis of protein extracts from HEK293T cells cotransfected with HA-K-Ras (WT, G12V, or Q61L) and Flag-Ephexin1. Immunoprecipitation was performed with a HA antibody, and the interaction of Ephexin1 to K-Ras was investigated by western blot using anti-Flag antibody. **b** A Raf1-RBD pulldown assay followed by western blot analysis with the indicated antibodies to measure the active GTP-bound forms of Ras in HEK293T cells transfected with either control siRNA or Ephexin1 siRNA. **c** A Raf1-RBD pulldown assay followed by western blot analysis with the indicated antibodies to detect Ras activation in HEK293T cells transfected with increasing amounts of Flag-tagged Ephexin1 expression plasmid as indicated. The results shown represent three independent experiments. **d** Control shRNA-transfected H1299 cells were transiently transfected either control or HA-K-Ras^G12V^, and the levels of B-Raf, pSer445 B-Raf, C-Raf, and pSer338 C-Raf were analyzed by western blotting. **e** Western blot analysis of phosphorylation of B-Raf, and C-Raf in control and Ephexin1 depleted HCT116 and H1299 cells treated with EGF (100 ng/ml) for the indicated amounts of time after 16 h of serum starvation. **f** Control and Ephexin1-depleted HEK293T cells were treated with or without EGF. 15 min after treatment, lysates of the whole cell extracts, cytosolic faction and light membrane faction were immunoblotted with indicated antibodies. **g** Lysates from control and Ephexin1-depleted HEK293T cells transfected with Flag-tagged B-Raf along with HA-tagged WT K-Ras or HA-tagged K-Ras^G12V^ were immunoprecipitated with anti-Flag antibody and subjected to western blot analysis with indicated antibodies. **h** Lysates from control and Ephexin1-depleted HEK293T cells transfected with HA-tagged WT K-Ras or HA-tagged K-Ras^G12V^ were immunoprecipitated with anti-HA antibody and subjected to western blot analysis with indicated antibodies. **i** Lysates from control and Ephexin1-depleted HEK293T cells transfected with Flag-tagged C-Raf along HA-tagged WT K-Ras or HA-tagged K-Ras^G12V^ were immunoprecipitated with anti-Flag antibody and subjected to western blot analysis with indicated antibodies.


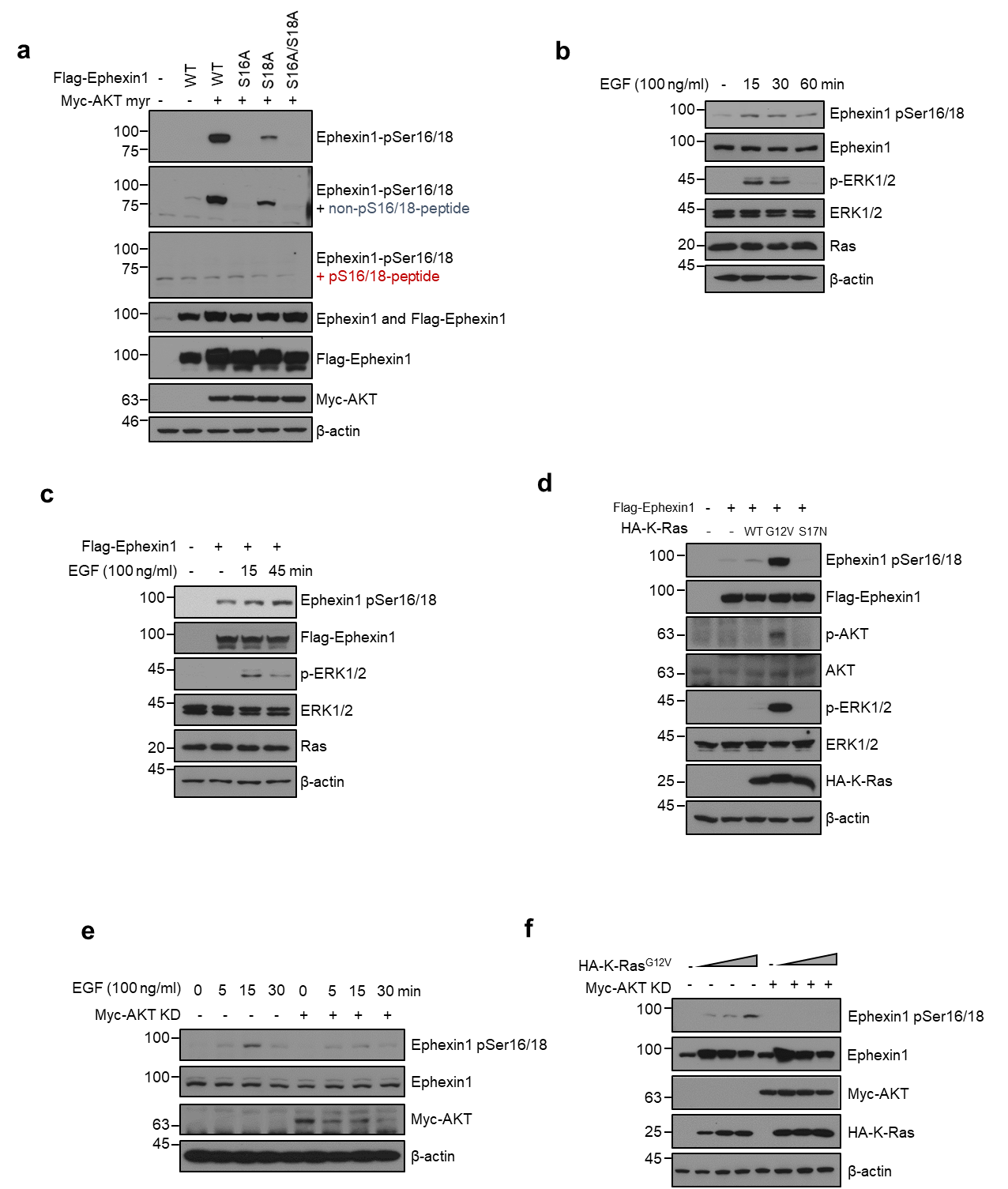


**Supplementary Fig. S7 EGF and K-Ras^G12V^ induce phosphorylation of Ephexin1 at Ser16/18. a** Rabbit polyclonal anti-pSer16/18-Ephexin1 antibody specifically recognizes pSer16/18-Ephexin1. Western blot analysis of HEK293T cells performed 48 h after transfection with Flag-tagged Ephexin1 WT, Ephexin1 S16A, Ephexin1 S18A, or Ephexin1 S16A/S18A together with Myc-myr-Akt. Experiments were carried out using an antibody against the rabbit polyclonal anti-pSer16/18-Ephexin1 antibody in the presence of either a control non-phosphorylated peptide or a phosphorylated peptide. **b** Western blot analysis using anti-pSer16/18-Ephexin1 antibody to detect phosphorylation of Ephexin1 at Ser16 and Ser18 in HEK293T cells treated with EGF (100 ng/ml) for the indicated amounts of time. **c** Western blot analysis using anti-pSer16/18-Ephexin1 antibody in Flag-tagged Ephexin1-transfected HEK293T cells treated with EGF (100 ng/ml) for the indicated amounts of time. **d** Western blot analysis using anti-pSer16/18-Ephexin1 antibody of HEK293T cells cotransfected with Flag-tagged Ephexin1 and WT or mutant HA-tagged K-Ras. **e** Western blot analysis for phosphorylation of Ephexin1 at Ser16 and Ser18 in control and Myc-tagged Akt-KD-transfected HEK293T cells treated with EGF (100 ng/ml) for the indicated amounts of time. **f** Western blot analysis for phosphorylation of Ephexin1 at Ser16 and Ser18 in HEK293T cells transfected with or without Myc-tagged Akt-KD along with increasing amounts of HA-tagged K-Ras^G12V^.

**
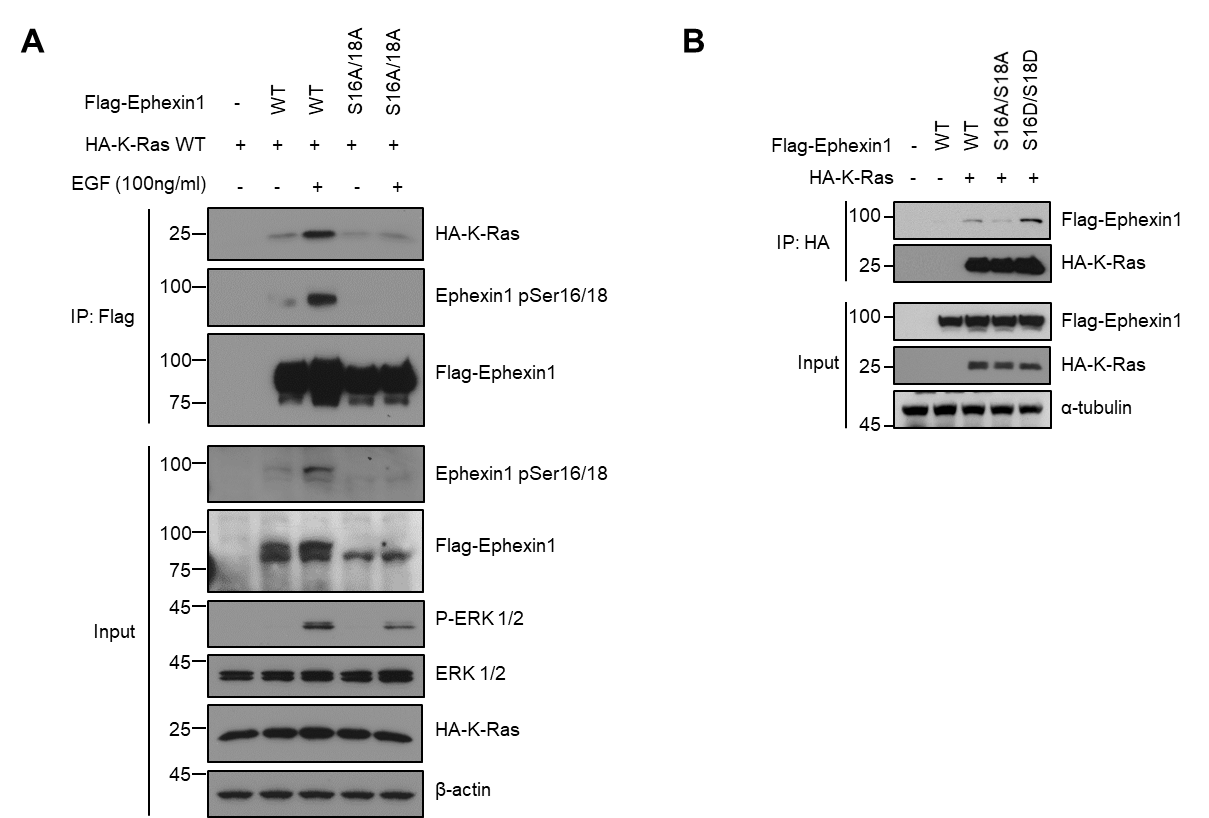
**

**Supplementary Fig. S8 Phosphorylation of Ephexin1 at serine 16/18 increases binding to K-Ras by EGF treatment.** **a** Co-immunoprecipitation analysis was conducted in HEK293T cells cotransfected with HA-tagged K-Ras^WT^ along with Flag-tagged WT Ephexin1, or Flag-tagged double phosphomutant Ephexin1 (S16A/S18A). Cells were then treated with or without EGF (100 ng/ml) after 16 h serum starvation. Immunoprecipitation was performed with a Flag antibody, and the interaction of Ephexin1 to K-Ras was investigated by western blot. **b** Co- immunoprecipitation analysis was conducted in HEK293T cells co-transfected with HA-K-Ras along with Flag-Ephexin1 (WT, S16A/S18A, S16D/S18D). Cell lysates were immunoprecipitated with anti-HA antibody, and the interaction of K-Ras to Ephexin1 were investigated by western blot.


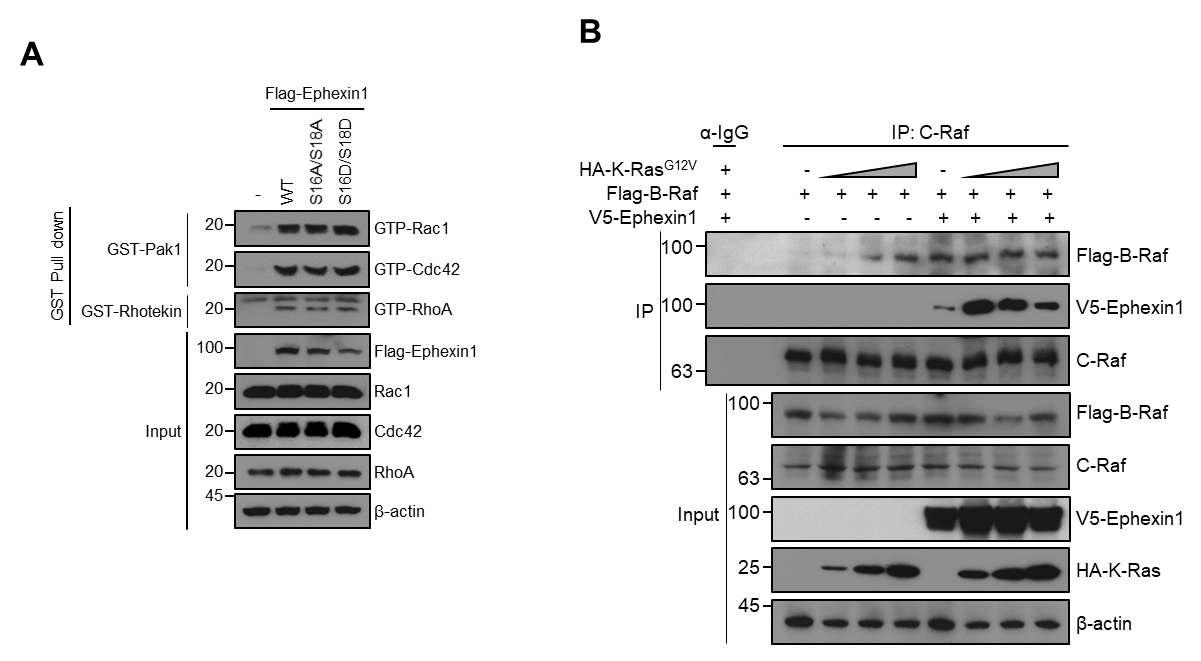


**Supplementary Fig. S9 Phosphorylation of Ephexin1 at Ser16/18 enhances the Raf/MEK/ERK signaling pathway. a** Phosphorylation of Ephexin1 at Ser16/18 does not contribute to the activation of RhoA, Cdc42, or Rac1. A GST pulldown assay, followed by western blotting with the indicated antibodies was performed on HEK293T cells transfected with Flag-tagged WT Ephexin1, S16A/S18A Ephexin1, or S16D/S18D Ephexin1 expression plasmids as indicated. Active GTP-bound forms of Rac1 and Cdc42 were analyzed by GST-Pak1-PBD pulldown and active GTP-bound forms of RhoA were analyzed by GST-Rhotekin-RBD pulldown. **b** Co-IP analysis of protein extracts from control and V5-Ephexin1-expressing HEK293T cells transfected with Flag-tagged B-Raf along with increasing amounts of HA-tagged K-Ras^G12V^. IP was performed with anti-C-Raf antibody, and the interaction of C-Raf to B-Raf was investigated by western blot using anti-Flag antibody.


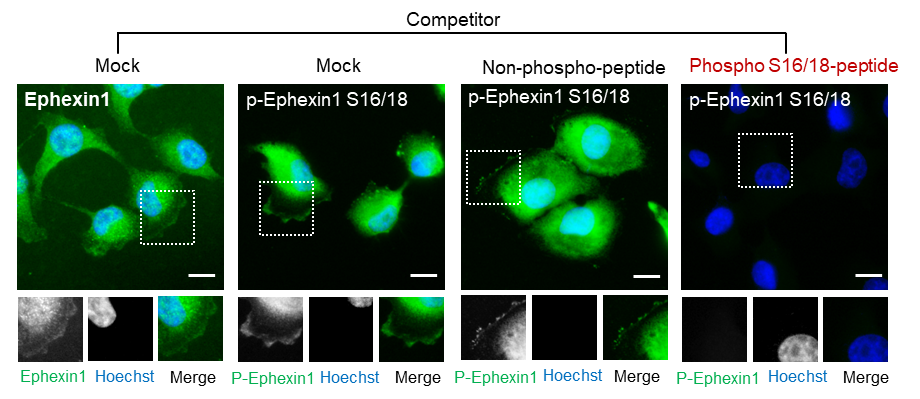


**Supplementary Fig. S10 Rabbit polyclonal anti-pSer16/18-Ephexin1 antibody specifically recognizes pSer16/18-Ephexin1.** Immunofluorescent staining with the polyclonal pSer16/18-Ephexin1 antibody (green) of fixed H1299 cells preincubated with control (mock), non-phosphorylated Ser16/18 peptide, or phospho-S16/18 peptide. Nuclei were stained with Hoechst 33258 (blue). Scale bars = 20 μm.

**Supplementary Table S1. List of primer sequences for cloning.**


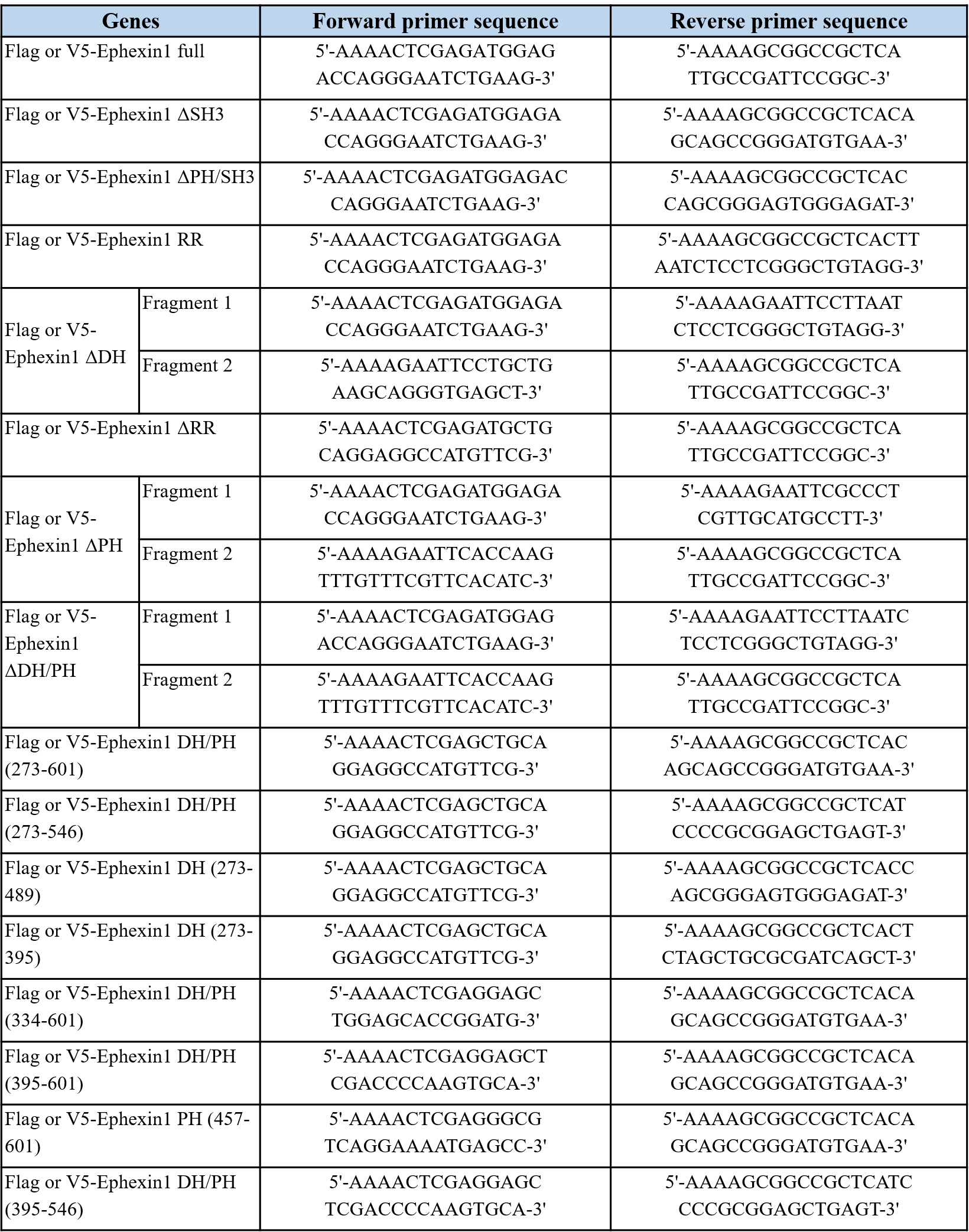


**Supplementary Table S2. List of primer sequences for site directed mutagenesis.**


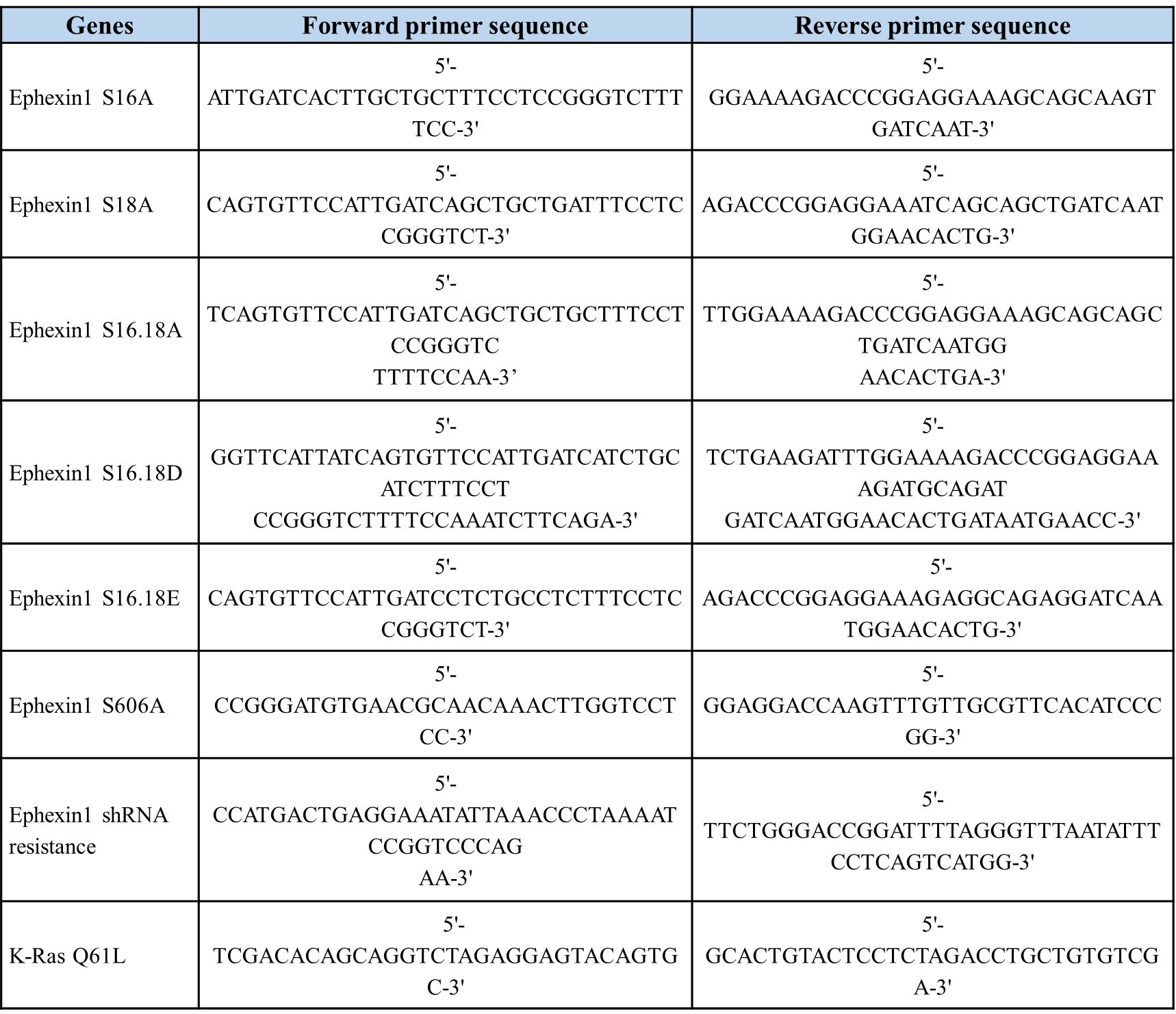


**Supplementary Table S3. List of primer sequences for RT-qPCR.**


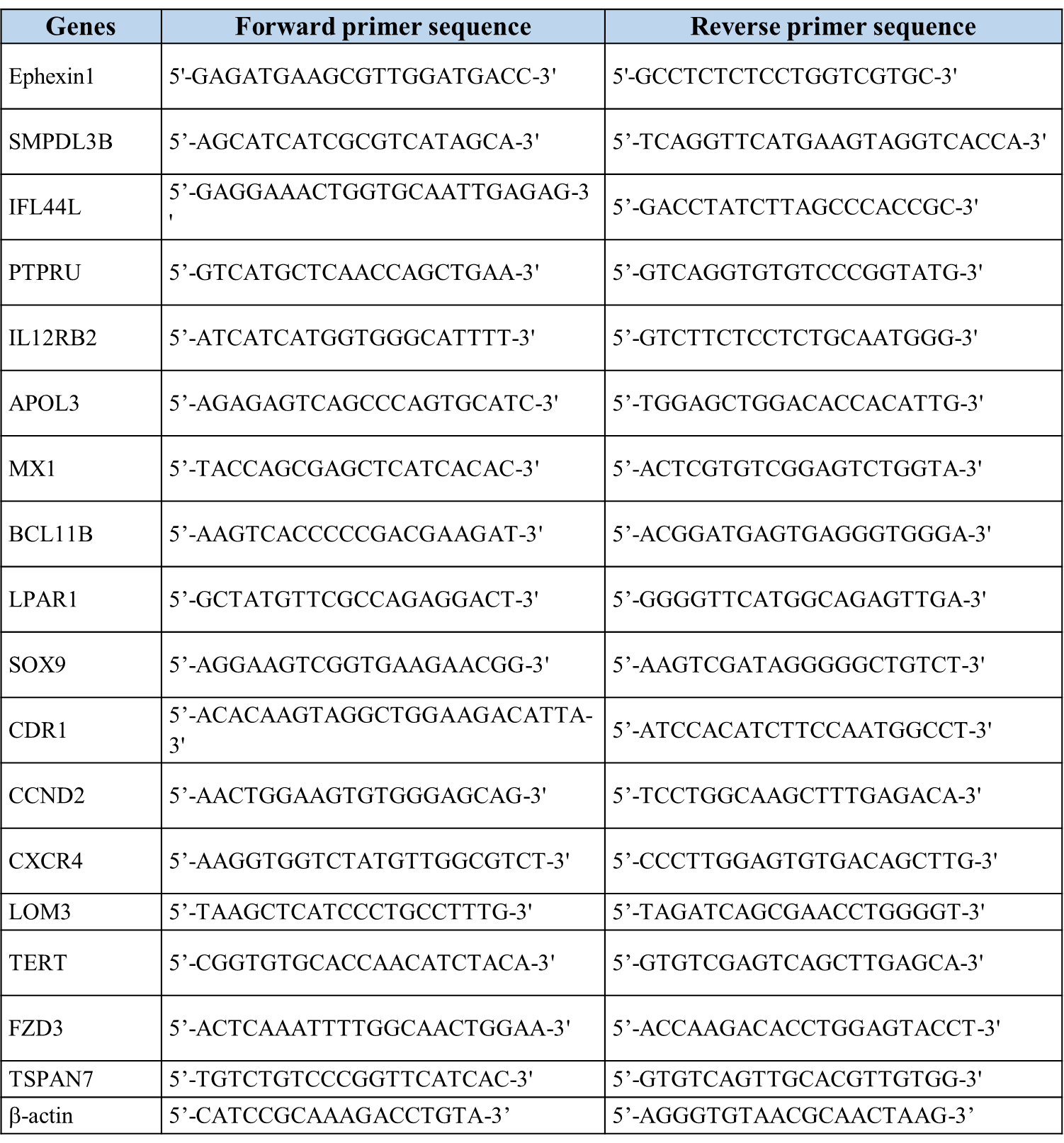


**Supplementary Table S4. List of sequences for RNAi**


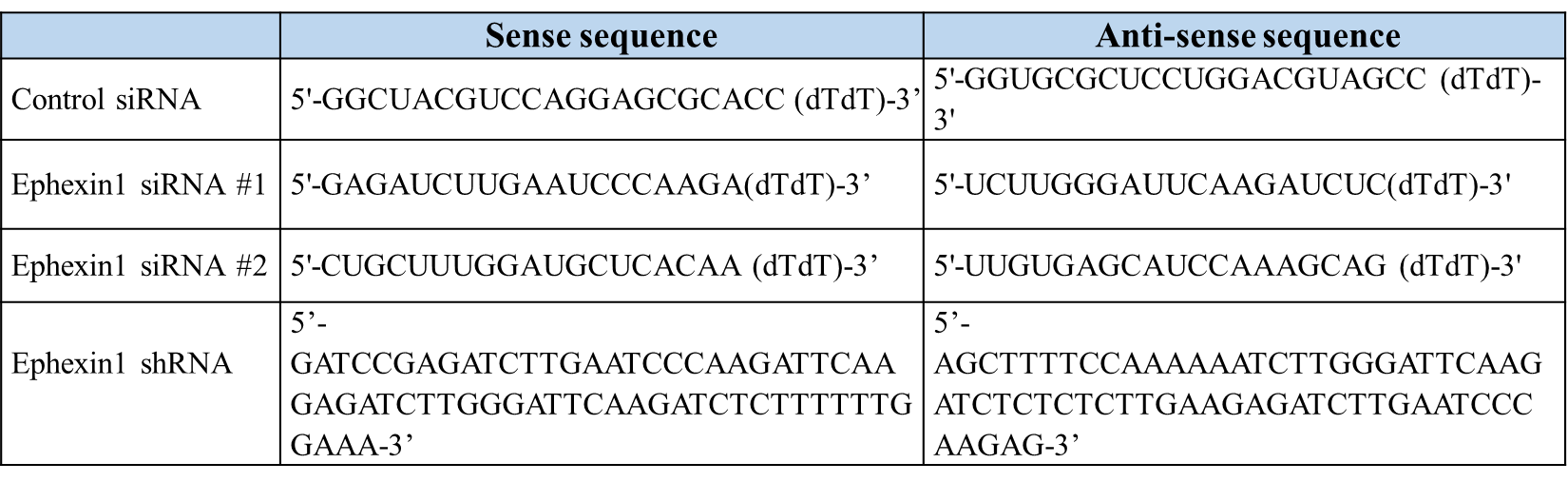


**Supplementary Table S5. List of antibodies**


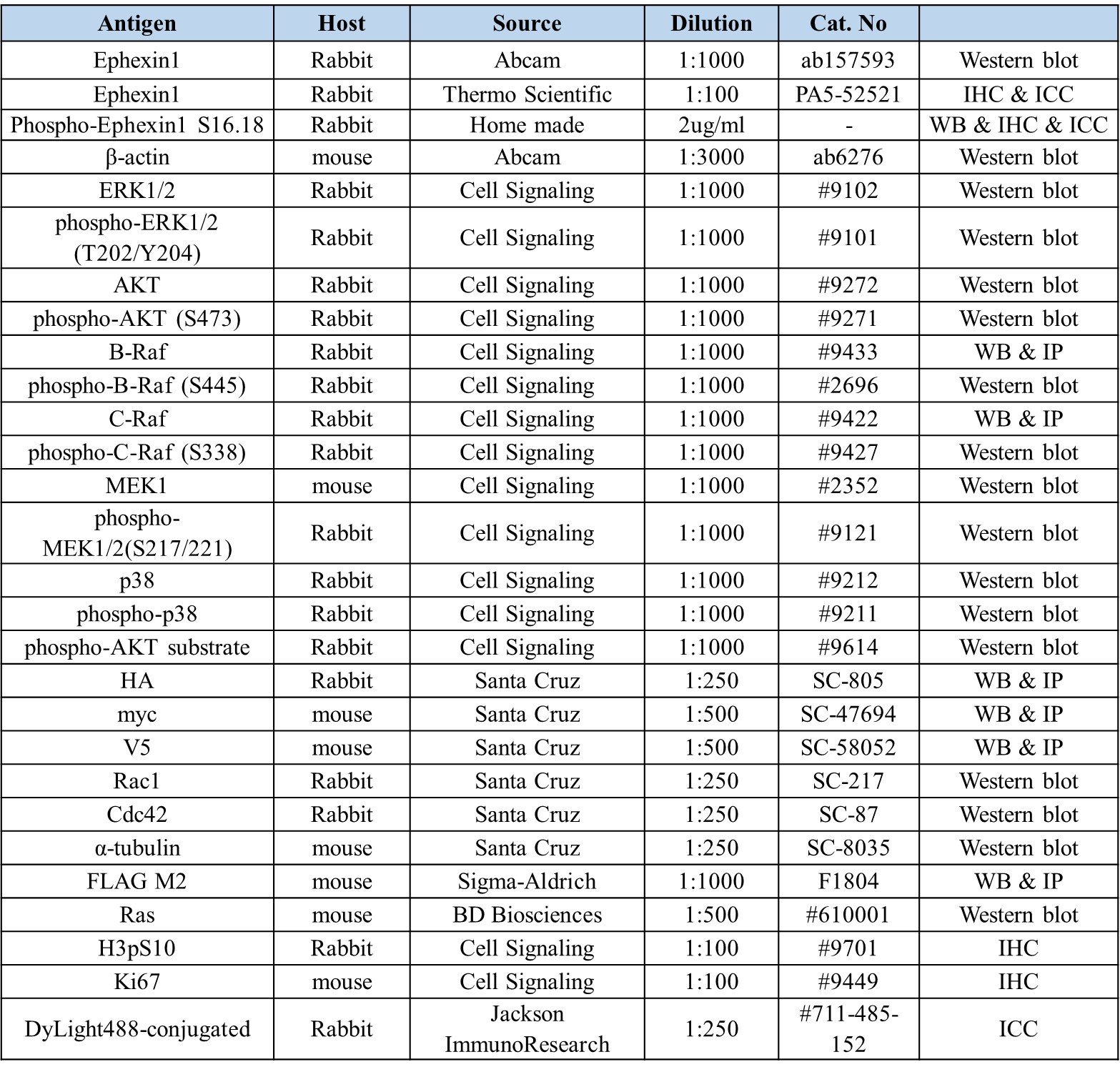


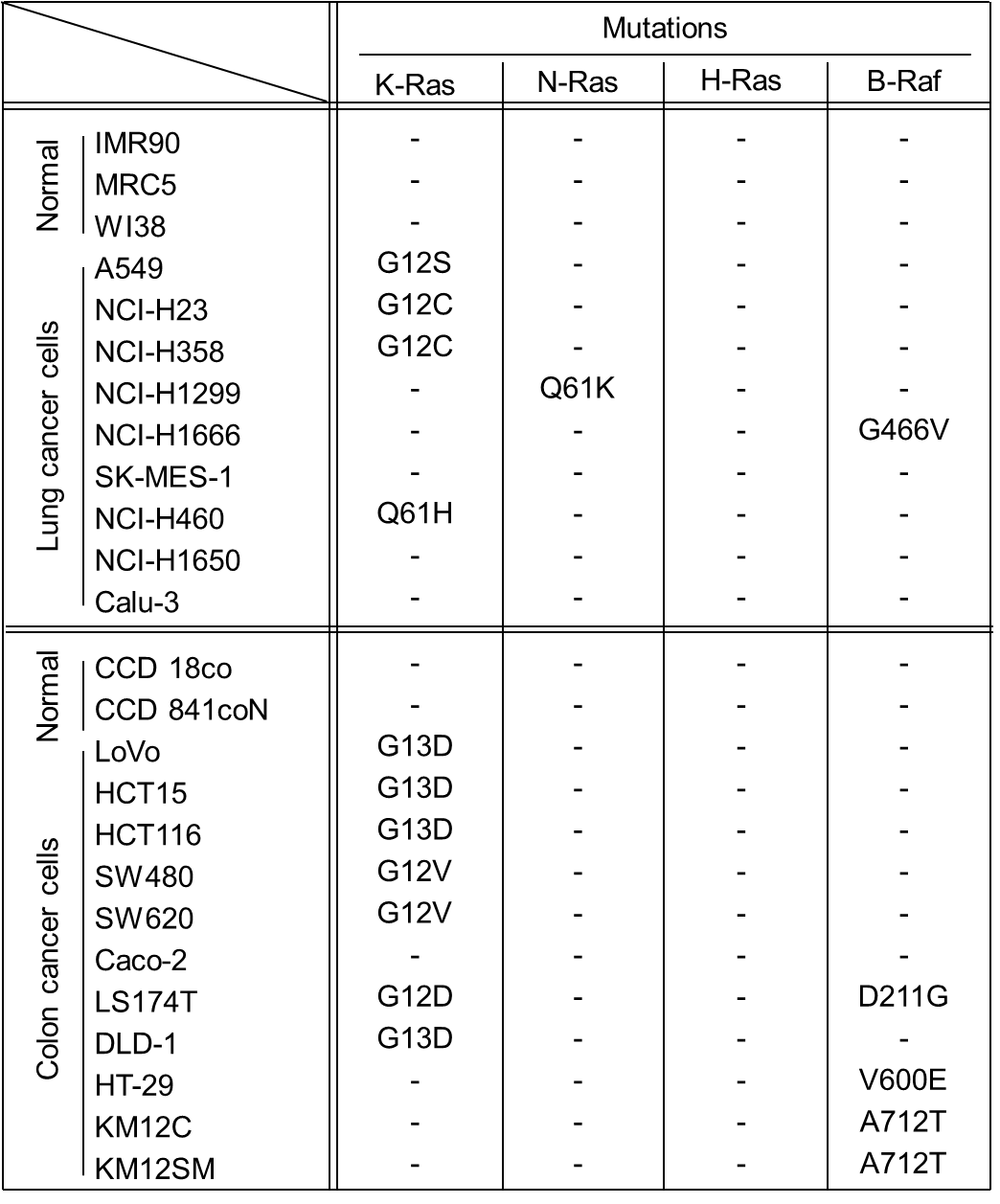
**Supplementary Table S6. Oncogenic Ras mutation status in cells used in this study.**
